# Weighted single-step genomic best linear unbiased prediction integrating variants selected from sequencing data by association and bioinformatics analyses

**DOI:** 10.1186/s12711-020-00568-0

**Published:** 2020-08-14

**Authors:** Aoxing Liu, Mogens Sandø Lund, Didier Boichard, Emre Karaman, Bernt Guldbrandtsen, Sebastien Fritz, Gert Pedersen Aamand, Ulrik Sander Nielsen, Goutam Sahana, Yachun Wang, Guosheng Su

**Affiliations:** 1 Center for Quantitative Genetics and Genomics, Aarhus University, 8830 Tjele, Denmark; 2 INRAE, AgroParisTech, GABI, Université Paris-Saclay, 78350 Jouy-en-Josas, France; 3 Nordic Cattle Genetic Evaluation, 8200 Aarhus N, Denmark; 4 Seges, 8200 Aarhus N, Denmark; 5 Key Laboratory of Animal Genetics, Breeding and Reproduction, MARA; National Engineering Laboratory for Animal Breeding, College of Animal Science and Technology, China Agricultural University, 100193 Beijing, P.R. China; 6 ALLICE, 75012 Paris, France

## Abstract

**Background:**

Sequencing data enable the detection of causal loci or single nucleotide polymorphisms (SNPs) highly linked to causal loci to improve genomic prediction. However, until now, studies on integrating such SNPs using a single-step genomic best linear unbiased prediction (ssGBLUP) model are scarce. We investigated the integration of sequencing SNPs selected by association (1262 SNPs) and bioinformatics (2359 SNPs) analyses into the currently used 54K-SNP chip, using three ssGBLUP models which make different assumptions on the distribution of SNP effects: a basic ssGBLUP model, a so-called featured ssGBLUP (ssFGBLUP) model that considered selected sequencing SNPs as a feature genetic component, and a weighted ssGBLUP (ssWGBLUP) model in which the genomic relationship matrix was weighted by the SNP variances estimated from a Bayesian whole-genome regression model, with every 1, 30, or 100 adjacent SNPs within a chromosome region sharing the same variance. We used data on milk production and female fertility in Danish Jersey. In total, 15,823 genotyped and 528,981‬ non-genotyped females born between 1990 and 2013 were used as reference population and 7415 genotyped females and 33,040 non-genotyped females born between 2014 and 2016 were used as validation population.

**Results:**

With basic ssGBLUP, integrating SNPs selected from sequencing data improved prediction reliabilities for milk and protein yields, but resulted in limited or no improvement for fat yield and female fertility. Model performances depended on the SNP set used. When using ssWGBLUP with the 54K SNPs, reliabilities for milk and protein yields improved by 0.028 for genotyped animals and by 0.006 for non-genotyped animals compared with ssGBLUP. However, with the SNP set that included SNPs selected from sequencing data, no statistically significant difference in prediction reliability was observed between the three ssGBLUP models.

**Conclusions:**

In summary, when using 54K SNPs, a ssWGBLUP model with a common weight on the SNPs in a given region is a feasible approach for single-trait genetic evaluation. Integrating relevant SNPs selected from sequencing data into the standard SNP chip can improve the reliability of genomic prediction. Based on such SNP data, a basic ssGBLUP model was suggested since no significant improvement was observed from using alternative models such as ssWGBLUP and ssFGBLUP.

## Background

A large number of causal loci or single nucleotide polymorphisms (SNPs) highly linked to causal loci have been discovered from sequencing data through association analyses [1, 2] and bioinformatics analyses [3, 4]. However, many of these SNPs are not part of the standard SNP chips that are commonly used in routine genomic evaluation, e.g. the Illumina Bovine SNP50 chip. By combining SNPs selected from sequencing data with those on the 54K bovine chip, improved reliabilities of genomic predictions for genotyped animals have been reported in various dairy cattle populations, such as Nordic Holsteins [5], Nordic Red [6], and Danish Jersey [7]. Simulations [8] showed that the accuracy of genomic prediction with a single-step genomic best linear unbiased prediction (ssGBLUP) model increased from 0.49 to 0.53 after integrating all the causal variants (i.e., simulated QTL), which were not included in the basic 60,000-SNP panel. In US Holsteins, with a ssGBLUP model, the prediction reliability for stature increased from 0.753 to 0.760 after integrating 16,648 SNPs selected from sequencing data by association analyses for 33 traits into the 54K-SNP chip [9].

One of the critical factors that impacts the performance of genomic prediction is the statistical model used. The GBLUP and ssGBLUP models assume that all SNPs have effects, which are drawn from a normal distribution assuming they all have the same variance [10–12], whereas Bayesian alphabet models assume that SNP effects are drawn from more or less complex mixture distributions and therefore can better accommodate SNPs with large effects [13–15]. Bayesian alphabet models usually outperform or are as good as the GBLUP model. However, the GBLUP model is still used in routine genomic evaluation because of its lower computational cost and simpler parameterization compared to Bayesian alphabet models. One strategy to achieve the strengths of both Bayesian alphabet and GBLUP models is to place emphasis on the most relevant markers that are discovered with Bayesian alphabet models when constructing the $\mathbf{G}$ matrix for GBLUP models, therefore, each trait can have its specific $\mathbf{G}$ matrix [16]. Many efforts have been put to find the optimal weighting strategy for constructing the weighted $\mathbf{G}$ matrix. For example, Su et al. [17] compared different weighting strategies for GBLUP models and suggested the use of the posterior SNP variance from the Bayesian R model [15] as the weighting factor and a common weight shared by a group of 30 adjacent SNPs when using the 54K-SNP chip. Similarly, Zhang et al. [18] compared different weighting strategies for ssGBLUP models using simulated datasets and reported that a common weight for a group of 20 adjacent SNPs achieved the highest reliability among all weighting strategies.

Integrating SNPs selected from sequencing data into the currently used 54K-SNP chip has the potential to include causal loci or SNPs in high linkage disequilibrium with causal loci. Therefore, a model that allows the inclusion of some SNPs with larger effects would be closer to the real distribution of SNP effects. For protein content in French dairy goats, which is a quantitative trait influenced by a major gene (explaining 40% of the genetic variance), the increase in accuracy was up to 6 percentage points from using a weighted ssGBLUP model compared to a basic ssGBLUP model when integrating the genotypes of the major gene into the genotype data of the 54K-SNP chip [19]. For stature in US Holsteins, when integrating 16,648 SNPs selected from sequencing data by association analyses, the prediction reliability decreased slightly from using a weighted ssGBLUP model with weights derived from the squared SNP effects compared to using a basic ssGBLUP model [9]. However, to date, for SNPs preselected from sequencing data by bioinformatics analyses (e.g., functional annotation), no comparison between a basic ssGBLUP and a weighted ssGBLUP model has been reported. In addition, placing emphasis on SNPs selected from sequencing data in genomic prediction models could be achieved by considering SNPs on the standard SNP chip and SNPs selected from sequencing data as two separate genetic components, i.e. a so-called featured GBLUP/ssGBLUP model. Results from a previous study on genotyped Danish Jersey animals showed that such a featured GBLUP model outperformed a basic GBLUP model for milk yield when integrating SNPs selected from sequencing data by association analyses and bioinformatics analyses, but no difference was observed between the two models for fat and protein yields, mastitis, and female fertility index [7]. To date, there is no study on genomic prediction using such a featured ssGBLUP model.

Our primary objective was to investigate the effects of integrating SNPs selected from sequencing data in genomic prediction of Danish Jersey cattle using ssGBLUP models. In addition, we compared a basic ssGBLUP model with alternative weighted/featured ssGBLUP models. Furthermore, for the ssWGBLUP model, we investigated the optimal region size for selecting region-specific weights.

## Methods

### Data

#### SNPs selected from sequencing data

In this study, we used two sets of SNPs selected from sequencing data included in the EuroGenomics customized Illumina Bovine low-density chip (customized LD, Illumina, Inc.) [20]. One SNP set included 1443 trait-associated SNPs selected from association analyses based on imputed full-sequencing information in bulls of three major dairy breeds of Denmark-Finland-Sweden (DFS) [6]. The other SNP set included 2618 SNPs selected from association analyses and bioinformatics analyses (within genes with a strong effect predicted from the Variant Effect Predictor (VEP) [21], within regulatory regions of genes, and breakpoints of structural variants) in five major dairy breeds from France (FRA) [20]. Details on the selection of DFS and FRA SNPs are in Boichard et al. [20] and Liu et al. [7]. For both DFS and FRA SNPs, the discovery populations used to preselect SNPs from sequencing data were not used in genomic prediction to avoid a false positive conclusion.

#### Genotype imputation

In total, 5480 Danish Jersey bulls, 1161 US Jersey bulls, and 48,039 Danish Jersey cows were genotyped with versions 1, 2, or 3 of the Illumina Bovine SNP50 chip (54K, Illumina, Inc.), the standard Bovine low-density Chip (standard LD, Illumina, Inc.) [22], or the EuroGenomics customized LD chip [20]. The number of animals genotyped with each of these SNP panels is in Table 1. Animals genotyped with different versions of SNP chips were imputed to 54K + DFS + FRA SNPs directly [23], using a family- and population-based approach implemented in the FImpute software [24]. The imputation accuracies of the DFS and FRA SNPs were assessed by randomly masking 10% of the animals genotyped with the customized LD chip to the standard LD chip, while the accuracy for the imputation from the standard LD chip to the 54K-SNP chip was assessed by randomly masking 10% of the animals genotyped with the 54K-SNP chip to the standard LD chip. The SNP-wise imputation accuracy was measured as the Pearson correlation between imputed and observed genotypes (coded as 0, 1, or 2) and the proportion of correctly imputed genotypes to all imputed genotypes (i.e., concordance rate). Only autosomal SNPs with a minor allele frequency higher than 0.01 and an imputation accuracy with both correlation and concordance rate higher than 0.8 were retained for genomic prediction. Ultimately, 37,074 SNPs from the 54K-SNP chip, 1262 DFS SNPs, and 2359 FRA SNPs were used for genomic prediction, with 28 SNPs shared by the DFS and FRA SNP sets. Regarding the imputation accuracy of these 40,667 SNPs, correlations between imputed and observed genotypes were 96.4% for the FRA SNPs, 96.6% for the DFS SNPs, and 97.0% for the imputation from the standard LD chip to the 54K SNPs, whereas concordance rates were 98.0% for the FRA SNPs, 98.3% for the DFS SNPs, and 98.4% for the imputation from the standard LD chip to the 54K SNPs.

**Table 1:** Number of animals genotyped with each SNP panel

SNP panel	Danish bulls	Danish cows	US bulls	Total
Standard low-density chip	7	14,012	–	14,019
EuroGenomics customized low-density chip	1824	33,027	–	34,851
54K-SNP chip	3490	998	1161	4488
54K-SNP chip and EuroGenomics customized low-density chip	159	2	–	161
Total	5480	48,039	1161	–

#### Phenotype

In order to investigate traits with various genetic architectures, three milk production traits including milk, fat, and protein yields and three female fertility traits including the interval from first to last insemination in heifers (IFLh) and cows (IFLc), and the interval from calving to first insemination (ICF) were analysed. Phenotypic data were provided by Nordic Cattle Genetic Evaluation (Aarhus, Denmark). For milk, fat, and protein yields, corrected 305-day yields [25], which were compiled from the data used in routine test-day evaluations of Nordic dairy cattle [26], were available for the first three lactations. For IFLh, raw field records were available for heifers. For IFLc and ICF, raw field records were available for the first three lactations. Details on trait definitions and data editing for the female fertility traits are in the report on routine genetic evaluation of Nordic Dairy cattle [26].

### Model

Breeding values were predicted by using single-trait models, including a pedigree-based BLUP (PBLUP) model, a basic single-step genomic BLUP (ssGBLUP) model, a so-called featured ssGBLUP (ssFGBLUP) model, and a weighted ssGBLUP (ssWGBLUP) model.

#### PBLUP

In the PBLUP model, the relationship matrix for the mixed model equation [27] was constructed using only pedigree information.

The model for IFLh is:

1 $${\mathbf{y}} = {\mathbf{X}}{\varvec{\upbeta}} + {\mathbf{Z}}_{{\mathbf{a}}} {\mathbf{a}} + {\mathbf{Z}}_{{{\mathbf{hys}}}} {\mathbf{hys}} + {\mathbf{e}},$$

and the model for milk, fat, and protein yields, IFLc, and ICF is:

2 $${\mathbf{y}} = {\mathbf{X}}{\varvec{\upbeta}} + {\mathbf{Z}}_{{\mathbf{a}}} {\mathbf{a}} + {\mathbf{Z}}_{{{\mathbf{pe}}}} {\mathbf{pe}} + {\mathbf{Z}}_{{{\mathbf{hys}}}} {\mathbf{hys}} + {\mathbf{e}},$$

where $\mathbf{y}$ is the vector of response variables (corrected 305-day yield for milk production traits, and raw phenotypes for female fertility traits); ${\varvec{\upbeta}}$ is the vector of fixed effects, including herd, year-season of insemination (for IFLh and IFLc), or year-season of calving (for milk, fat, and protein yields, and ICF), calving age expressed in months (for milk production traits) or age at first insemination expressed in months (for female fertility traits), and parity (for all traits except for IFLh); $\mathbf{a}$ is the vector of additive genetic effects; $\mathbf{p}\mathbf{e}$ is the vector of permanent environmental effects; $\mathbf{h}\mathbf{y}\mathbf{s}$ is the vector of random effects of herd-year-season of insemination (for IFLh and IFLc) or herd-year-season of calving (for milk, fat, protein, and ICF); $\mathbf{e}$ is the vector of random residual effects; and $\mathbf{X}$, ${\mathbf{Z}}_{\mathbf{a}}$, ${\mathbf{Z}}_{\mathbf{p}\mathbf{e}}$, and ${\mathbf{Z}}_{\mathbf{h}\mathbf{y}\mathbf{s}}$ are incidence matrices relating ${\varvec{\upbeta}}$, $\mathbf{a}$, $\mathbf{p}\mathbf{e}$ and $\mathbf{h}\mathbf{y}\mathbf{s}$ to $\mathbf{y}$. It is assumed that $\mathbf{a}\boldsymbol{ }\sim \boldsymbol{ }N(0,\mathbf{A}{\upsigma }_{\mathrm{a}}^{2})$, $\mathbf{p}\mathbf{e}\boldsymbol{ }\sim \boldsymbol{ }N(0,\mathbf{I}{\upsigma }_{pe}^{2})$, $\mathbf{h}\mathbf{y}\mathbf{s}\boldsymbol{ }\sim \boldsymbol{ }N(0,\mathbf{I}{\upsigma }_{hys}^{2})$, and $\mathbf{e}\sim \boldsymbol{ }N(0,\mathbf{I}{\upsigma }_{e}^{2})$. $\mathbf{A}$ is an additive relationship matrix that considers inbreeding, based on a pedigree (without genetic group) including 9595 males and 656,389 females, which was constructed by tracing the animals with phenotypes three generations back. The estimation of the variance components, and the prediction of breeding values with the PBLUP models were performed using the DMU software [28].

#### ssGBLUP

In the ssGBLUP model, the relationship matrix for the mixed model equation was constructed using both pedigree (without genetic group) and genomic information. The pedigree used for the ssGBLUP model was exactly the same as that used for the PBLUP model. The effects in the ssGBLUP model are also exactly the same as those in the above PBLUP model, but this model assumes $\mathbf{a}\boldsymbol{ }\sim \boldsymbol{ }N(0,\mathbf{H}{\upsigma }_{\mathrm{a}}^{2})$, where $\mathbf{H}$ is a unified genetic relationship matrix with ${\mathbf{H}}^{-1}$ constructed as [10, 11]:

$${\mathbf{H}}^{ - 1} = {\mathbf{A}}^{ - 1} + \left[ {\begin{array}{*{20}c} 0 & 0 \\ 0 & {{\mathbf{G}}^{ - 1} - {\mathbf{A}}_{22}^{ - 1} } \\ \end{array} } \right],$$

where $\mathbf{A}$ is the pedigree-based relationship matrix for both genotyped and non-genotyped animals, $\mathbf{G}$ is the genomic relationship matrix adjusted to the same scale as ${\mathbf{A}}_{22}$, and ${\mathbf{A}}_{22}$ is a subset of $\mathbf{A}$ for genotyped animals. Here, inbreeding was considered in all the relationship matrices. $\mathbf{G}$ was defined as:

$${\mathbf{G}} = \left( {1 - {\upomega }_{a} } \right){\mathbf{G}}_{{\varvec{a}}} + {\upomega }_{a} {\mathbf{A}}_{22} ,$$

where ${\upomega }_{a}$ is the fraction of the genetic variance not captured by SNPs and ${\mathbf{G}}_{{\varvec{a}}}$ is the adjusted genomic relationship matrix. In this study, ${\upomega }_{a}$ was set to 0.2 based on [29]. The ${\mathbf{G}}_{{\varvec{a}}}$ matrix was adjusted for the differences in scale between the original genomic relationship matrix ${\mathbf{G}}_{{\varvec{m}}}$ and the pedigree-based relationship matrix ${\mathbf{A}}_{22}$ as previously described [30].

$${\mathbf{G}}_{{\varvec{a}}} = {\upbeta }{\mathbf{G}}_{{\varvec{m}}} + {\alpha ,}$$

where $\mathrm{\alpha }$ and $\upbeta$ are derived from the following equations:

$$\begin{gathered} Avg\left( {diag\left( {{\mathbf{G}}_{{\varvec{m}}} } \right)} \right){\upbeta } + {\upalpha } = Avg\left( {diag\left( {{\mathbf{A}}_{22} } \right)} \right), \hfill \\ Avg\left( {{\mathbf{G}}_{{\varvec{m}}} } \right){\upbeta } + {\upalpha } = Avg\left( {{\mathbf{A}}_{22} } \right). \hfill \\ \end{gathered}$$

Furthermore, ${\mathbf{G}}_{{\varvec{m}}}$ was constructed using the following method [12]:

$${\mathbf{G}}_{{\varvec{m}}} = \frac{{{\mathbf{MDM^{\prime}}}}}{{\sum {2p_{i} q_{i} } }},$$

where $\mathbf{M}$ is a matrix with the elements in column $j$ being $0-2{p}_{j}$, $1-2{p}_{j}$, or $2-2{p}_{j}$ for genotypes ${A}_{1}{A}_{1}$, ${A}_{1}{A}_{2}$, or ${A}_{2}{A}_{2}$ respectively, ${p}_{j}$ is the allele frequency of ${A}_{2}$ at locus $j$ computed from all genotyped animals in the model, and $\mathbf{D}$ is an identity matrix. Prediction of breeding values with the ssGBLUP models was performed using the DMU software [28], but because of the extensive computational cost, this software cannot estimate variance components directly from the ssGBLUP models. Instead, the variance components estimated from the above PBLUP models that included both genotyped and non-genotyped reference animals (see Additional file 1: Table S1) were used to predict the breeding values with the ssGBLUP models. Thanks to this strategy, the variance components of a trait were the same for analyses using different SNP sets. Four SNP sets were used to construct the relationship matrices: (i) 54K (37,074 SNPs), (ii) 54K + DFS (38,336 SNPs), (iii) 54K + FRA (39,433 SNPs), or (iv) 54K + DFS + FRA (40,667 SNPs).

#### ssFGBLUP

In the ssFGBLUP model, two ${\mathbf{H}}^{-1}$ matrices were constructed, i.e. one by combining the 54K-SNP chip and the pedigree data, and one by combining the SNPs selected from the sequencing data and the pedigree data. When constructing the relationship matrices for the ssFGBLUP models, three SNP sets were used: (i) 54K + DFS, (ii) 54K + FRA, or (iii) 54K + (DFS + FRA).

The ssFGBLUP model for IFLh is:

3 $${\mathbf{y}} = {\mathbf{X}}{\varvec{\upbeta}} + {\mathbf{Z}}_{{\mathbf{a}}} {\mathbf{a}}_{{54{\mathbf{K}}}} + {\mathbf{Z}}_{{\mathbf{a}}} {\mathbf{a}}_{{{\mathbf{seq}}}} + {\mathbf{Z}}_{{{\mathbf{hys}}}} {\mathbf{hys}} + {\mathbf{e}},$$

and the ssFGBLUP model for milk, fat, and protein yields, IFLc, and ICF is:

4 $${\mathbf{y}} = {\mathbf{X}}{\varvec{\upbeta}} + {\mathbf{Z}}_{{\mathbf{a}}} {\mathbf{a}}_{{54{\mathbf{K}}}} + {\mathbf{Z}}_{{\mathbf{a}}} {\mathbf{a}}_{{{\mathbf{seq}}}} + {\mathbf{Z}}_{{{\mathbf{pe}}}} {\mathbf{pe}} + {\mathbf{Z}}_{{{\mathbf{hys}}}} {\mathbf{hys}} + {\mathbf{e}},$$

where $\mathbf{y}$, $\mathbf{X}$, ${\varvec{\upbeta}}$, ${\mathbf{Z}}_{\mathbf{h}\mathbf{y}\mathbf{s}}$, $\mathbf{h}\mathbf{y}\mathbf{s}$, and $\mathbf{e}$ are the same as for the above PBLUP models; ${\mathbf{a}}_{54\mathbf{K}}$ is the vector of additive genetic effects captured by the 54K SNPs and the pedigree; ${\mathbf{a}}_{\mathbf{s}\mathbf{e}\mathbf{q}}$ is the vector of additive genetic effects captured by the SNPs selected from sequencing data and the pedigree; and ${\mathbf{Z}}_{\mathbf{a}}$ is the incidence matrix relating ${\mathbf{a}}_{54\mathbf{K}}$ and ${\mathbf{a}}_{\mathbf{s}\mathbf{e}\mathbf{q}}$ to $\mathbf{y}$. It is assumed that ${\mathbf{a}}_{54\mathbf{K}}\sim N(0,{\mathbf{H}}_{54\mathbf{K}}{\upsigma }_{{\mathrm{a}}_{54\mathrm{K}}}^{2})$ and ${\mathbf{a}}_{\mathbf{s}\mathbf{e}\mathbf{q}}\sim N(0,{\mathbf{H}}_{\mathbf{s}\mathbf{e}\mathbf{q}}{\upsigma }_{{\mathrm{a}}_{\mathrm{seq}}}^{2})$, with no covariance being constructed between ${\mathbf{a}}_{54\mathbf{K}}$ and ${\mathbf{a}}_{\mathbf{s}\mathbf{e}\mathbf{q}}$. The prediction of breeding values with the ssFGBLUP models was performed using the DMU software [28], but because of the extensive computational cost, this software [28] cannot estimate variance components directly from the ssFGBLUP models. Instead, the total additive genetic variance (the sum of ${\upsigma }_{{\mathrm{a}}_{54\mathrm{K}}}^{2}$ and ${\upsigma }_{{\mathrm{a}}_{\mathrm{seq}}}^{2}$), ${\upsigma }_{\mathrm{pe}}^{2}$, and ${\upsigma }_{{\mathrm{a}}_{\mathrm{e}}}^{2}$ used to predict breeding values with the ssFGBLUP models were obtained from the above PBLUP models (see Additional file 1: Table S1), whereas the proportion of additive genetic variance explained by each genetic component was obtained from the FGBLUP model using genotyped animals in the reference population (see Additional file 1: Table S2).

#### ssWGBLUP

In the ssWGBLUP model, the genomic relationship matrix was weighted by the SNP variances estimated from a Bayesian whole-genome regression model, which used genotyped animals in the reference population. Here, four SNP sets were used to construct the relationship matrices: (i) 54K, (ii) 54K + DFS, (iii) 54K + FRA, or (iv) 54K + DFS + FRA. The effects of the ssWGBLUP model were exactly the same as those in the above ssGBLUP model, but the $j$th diagonal element of the $\mathbf{D}$ matrix (an identity matrix in ssGBLUP) used to construct the ${\mathbf{G}}_{{\varvec{m}}}$ matrix is the weight on the $j$th SNP. In this study, the weight on the $j$th SNP is its posterior variance (standardized by dividing by the average posterior variance over all SNPs) obtained from a Bayesian model that accounts for region-specific variances (hereafter, BayesN0) [31], where every 1, 30, or 100 adjacent SNPs (BayesN0_bin1, BayesN0_bin30, or BayesN0_bin100) on a chromosome region were assumed to form a region that shared the same variance. In order to investigate the advantage of a Bayesian model that accounts for region-specific variances over a model in which all SNPs share the same variance, a BayesN0 model that considers the whole genome as one region (BayesN0_WG, equivalent to GBLUP) was also used for genomic prediction.

The BayesN0 model used for all traits is:

$${\mathbf{y}} = \mathbf{1}\mu + \sum {\mathbf{M}}_{i} {\varvec{\upalpha}}_{i} + {\mathbf{e}},$$

where $\mathbf{y}$ is the vector of yield deviations (YD) for genotyped animals in the reference population; $\mathbf{1}$ is a vector of ones; $\mu$ is the overall mean; ${\varvec{\upalpha}}_{i}$ is the vector of SNP effects in the *i*th genomic region; ${\mathbf{M}}_{i}$ is the genotype matrix in the $i$th genomic region; and $\mathbf{e}$ is the vector of random residual effects. It is assumed that ${\varvec{\upalpha}}_{i}| {\upsigma }_{i}^{2}\sim {N}(0,{\upsigma }_{i}^{2})$ and ${\upsigma }_{i}^{2}\sim {v}_{\alpha }{S}_{\alpha }^{2}{\chi }_{{v}_{\alpha }}^{-2}$, where ${\upsigma }_{i}^{2}$ is the variance of the SNP effect for each SNP within the $i$th genomic region, ${v}_{\alpha }$ is the degree of freedom, and ${S}_{\alpha }^{2}$ is the scale parameter. It is assumed that $\mathbf{e}\sim N(0,\mathbf{R}{\upsigma }_{\mathrm{e}}^{2})$, where $\mathbf{R}$ is a diagonal matrix with elements ${d}_{jj}=(1-{r}_{YD}^{2})/{r}_{YD}^{2}$ to account for heterogeneous residual variances (${\upsigma }_{\mathrm{e}}^{2}$) due to different reliabilities of the YD (${r}_{YD}^{2}$). In this study, YD, which is defined as the sum of the estimated breeding values (EBV) and estimated residuals, i.e., the phenotype adjusted for all effects in the model other than the additive genetic effect, was derived from the above PBLUP model with phenotypes of animals in both the reference and validation populations using the DMU software [28]. Each of the BayesN0 models was run as a single Markov chain with a total length of 50,000 samples and the first 10,000 samples discarded as burn-in. Ultimately, every 20th sample of the remaining 40,000 samples was saved for the posterior analysis. The analyses with the BayesN0 models were performed using in-house scripts written in Julia language [32]. The prediction of breeding values with the ssWGBLUP models was performed using the DMU software [28]. The variance components used for predicting breeding values with the ssWGBLUP models were obtained from the above PBLUP models (see Additional file 1: Table S1) since the DMU software cannot estimate the variance components directly from the ssWGBLUP models because of the extensive computational cost.

#### Reliability and bias of prediction

For the prediction of breeding values, 15,823 genotyped and 528,981‬ non-genotyped females born between 1990 and 2013 were used as the reference population. To avoid close relationships between reference and validation populations, 7415 genotyped females and 33,040 non-genotyped females born between 2014 and 2016 that did not have any sib or daughter born before 2014 were used as the validation population. For each trait, the numbers of genotyped and non-genotyped animals in reference and validation populations are in Table 2. By using the young cows as the validation population, the overlap between the validation population and the discovery population of DFS SNPs was elaborately avoided since the discovery population of DFS SNPs consisted of bulls only. In addition, to avoid using the same information for selecting DFS SNPs and investigating them in prediction, the genotyped bulls were excluded from the reference population in the models used to predict breeding values (the PBLUP and three ssGBLUP models) and in the models used to derive weights (BayesN0 models) for the ssWGBLUP models. From the perspective of phenotypes, given that all the daughters with phenotypes, either genotyped or non-genotyped, have already been included, adding genotyped bulls will not add information for the PBLUP and three ssGBLUP models. However, in practice, a genetic evaluation based on a ssGBLUP model can include the genotypes of bulls when constructing the relationship matrix in order to benefit their non-genotyped daughters. The number of genotyped animals used in genomic prediction was smaller than that involved in genotype imputation since some young genotyped animals did not have any phenotypic record during the period of data collection. However, including these young genotyped animals in genotype imputation can benefit the imputation of SNPs selected from sequencing data for genotyped animals involved in genomic prediction, since most of the young animals were genotyped with the customized LD chip. The reliability of predictions was measured as the squared correlation between EBV and YD divided by the average reliability of YD in the validation population for genotyped and non-genotyped animals, separately. In the validation population, the average reliabilities of YD were consistent for genotyped and non-genotyped animals, and equal to 0.421 for milk yield, 0.308 for fat yield, 0.353 for protein yield, 0.014 for IFLh, 0.033 for IFLc, and 0.055 for ICF. Reliabilities of YD varied little among the validation animals since they comprised young females born between 2014 and 2016, with most of them (> 90% for milk production traits and > 70% for female fertility traits) having only one lactation record until the end of data collection. The bias of prediction was measured as the regression coefficient of YD on the EBV for genotyped and non-genotyped animals in the validation population separately. When calculating reliability and bias, unweighted methods (e.g. unweighted regression for bias) were used because of the small variation in reliability of YD among validation animals. A non-parametric bootstrapping procedure with 10,000 samples as described in Liu et al. [7] was used to test the difference in genomic prediction between models and between SNP sets. The Bonferroni correction was used to control the false positive rate resulting from multiple tests.

**Table 2:** Number of genotyped and non-genotyped animals in reference and validation populations

Reference/validation	Population	Milk, fat, and protein	IFLh	IFLc	ICF
Reference	Genotyped	14,645	9803	12,868	12,831
	Non-genotyped	413,727	216,532	405,057	405,121
Validation	Genotyped	4099	3749	3050	2833
	Non-genotyped	20,883	15,425	15,030	14,114

*IFLh* the interval from first to last insemination in heifers, *IFLc* the interval from first to last insemination in cows, *ICF* the interval from calving to first insemination

## Results

With the BayesN0_WG model (equivalent to GBLUP) (see Additional file 1: Table S3), reliabilities increased significantly after integrating all the SNPs selected from sequencing data compared to the use of the 54K SNP set only, for milk yield (0.045) and protein yield (0.023), but not for fat yield and female fertility traits. However, with the Bayesian models other than the BayesN0_WG model, no significant difference was observed between using the 54K SNP set and a set combining the 54K SNPs and the SNPs selected from sequencing data, except when the BayesN0_bin100 model was used for milk yield. Overall, reliabilities did not differ significantly between the three SNP sets that included SNPs selected from sequencing data, except the reliability obtained by using the 54K + DFS + FRA SNP set, which was 0.006 higher than that of the 54K + FRA SNP set averaged over three milk production traits using the BayesN0_WG model. Overall, reliabilities were the same for all region sizes, except for protein yield, for which a region size of 30 SNPs achieved higher reliability than a region size of 1 or 100 SNPs.

**Fig. 1:**
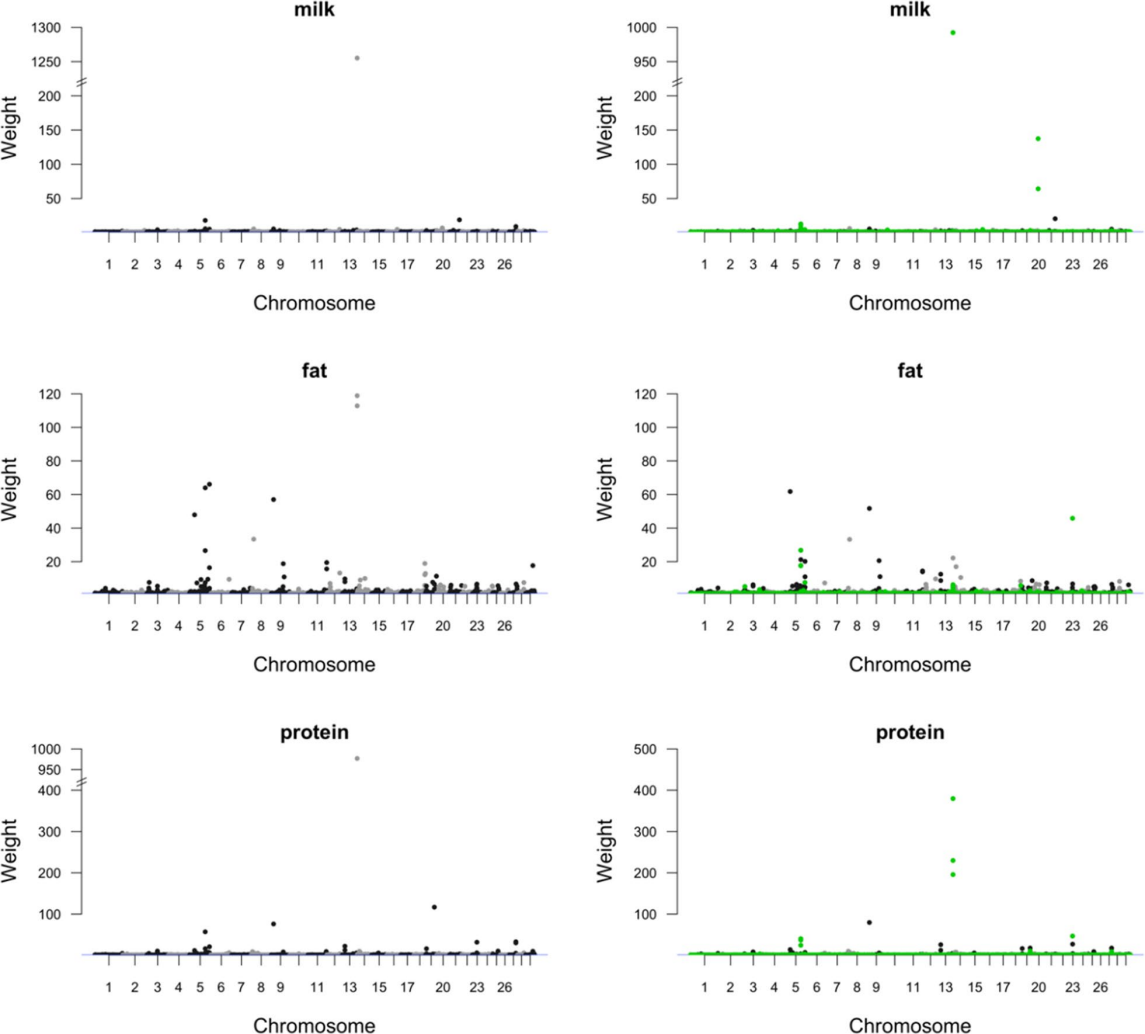
Weights generated from a BayesN0_bin1 model for milk, fat, and protein yields, using the 54K (left) and 54K + DFS + FRA (right) SNP sets. The SNPs from the DFS + FRA SNP set are highlighted with green colour

**Fig. 2:**
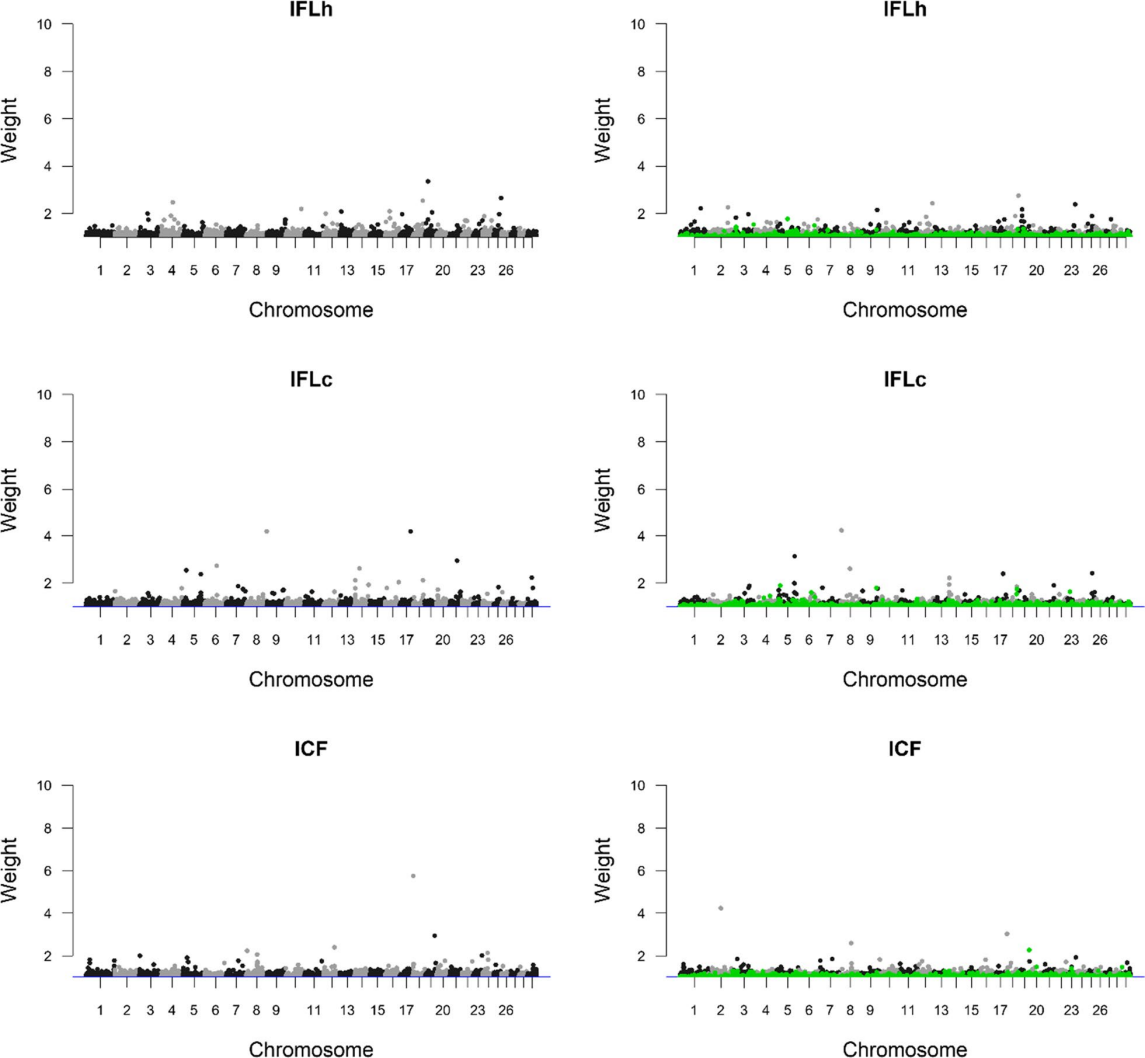
Weights generated from a BayesN0_bin1 model for the interval from first to last insemination in heifers (IFLh) and cows (IFLc), and the interval from calving to first insemination (ICF), using the 54K (left) and 54K + DFS + FRA (right) SNP sets. The SNPs from the DFS + FRA SNP set are highlighted with green colour

Figures 1 and 2 present the weights (standardized SNP variances) for the 54K and 54K + DFS + FRA SNP sets obtained by using a BayesN0_bin1 model. The average weight was equal to 1 for each analysis due to the standardization process. For milk production traits, the largest weight when using the 54K SNP set (1260 for milk yield, 119 for fat yield, and 977 for protein yield) was greater than that obtained when using the 54K + DFS + FRA SNP set (993 for milk yield, 62 for fat yield, and 380 for protein yield). For female fertility traits, the largest weight when using the 54K SNP set (3 for IFLh, 4 for IFLc, and 6 for ICF) was similar to that when using the 54K + DFS + FRA SNP set (3 for IFLh, 4 for IFLc, and 4 for ICF). Generally, a large proportion of the top SNPs for milk and protein yields were SNPs selected from sequencing data, whereas this was much less clear for fat yield and female fertility traits.

Reliabilities from the PBLUP and various ssGBLUP models for both genotyped and non-genotyped animals are in Tables 3 and 4. For all traits, ssGBLUP models outperformed PBLUP models. With the 54K SNP set, reliabilities improved from 0.156 with the PBLUP model to 0.366 with a basic ssGBLUP model for genotyped animals, and from 0.127 to 0.153 for non-genotyped animals, averaged over all traits. In addition, for genotyped animals, all ssGBLUP models outperformed the corresponding Bayesian models with the same bin size, due to the inclusion of performance information from non-genotyped females. The improvements of reliabilities ranged from 0.006 for protein yield to 0.112 for IFLh, averaged over all SNP sets.

**Table 3:** Reliabilities from a pedigree-based BLUP (PBLUP) model and different ssGBLUP models using different SNP sets for milk production traits

Trait	Population	Model	Pedigree	SNP			
				54K	54K + DFS	54K + FRA	54K + DFS + FRA
Milk	Genotyped	PBLUP	0.225	–	–	–	–
		ssGBLUP	–	_c_0.595^c^	_b_0.633^ab^	_b_0.631^b^	_a_0.637^a^
		ssFGBLUP	–	–	_ab_0.641^a^	_ab_0.630^a^	_a_0.637^a^
		ssWGBLUP_bin1	–	_a_0.641^a^	_ab_0.645^a^	_ab_0.642^a^	_a_0.645^a^
		ssWGBLUP_bin30	–	_a_0.640^b^	_a_0.648^a^	_a_0.644^ab^	_a_0.645^ab^
		ssWGBLUP_bin100	–	_b_0.627^b^	_ab_0.641^a^	_ab_0.639^a^	_a_0.640^a^
	Non-genotyped	PBLUP	0.200	–	–	–	–
		ssGBLUP	–	_b_0.240^b^	_b_0.247^a^	_a_0.247^a^	_a_0.248^a^
		ssFGBLUP	–	–	_ab_0.248^a^	_a_0.247^a^	_a_0.247^a^
		ssWGBLUP_bin1	–	_a_0.249^a^	_a_0.250^a^	_a_0.250^a^	_a_0.250^a^
		ssWGBLUP_bin30	–	_a_0.248^a^	_ab_0.249^a^	_a_0.249^a^	_a_0.249^a^
		ssWGBLUP_bin100	–	_a_0.247^b^	_ab_0.249^a^	_a_0.248^ab^	_a_0.248^ab^
Fat	Genotyped	PBLUP	0.125	–	–	–	–
		ssGBLUP	–	_a_0.391^ cd^	_ab_0.397^ab^	_a_0.391^bd^	_a_0.396^ac^
		ssFGBLUP	–	–	_a_0.405^a^	_a_0.387^b^	_a_0.398^a^
		ssWGBLUP_bin1	–	_a_0.383^a^	_b_0.387^a^	_a_0.382^a^	_a_0.386^a^
		ssWGBLUP_bin30	–	_a_0.386^b^	_ab_0.398^a^	_a_0.390^ab^	_a_0.396^ab^
		ssWGBLUP_bin100	–	_a_0.389^a^	_ab_0.386^a^	_a_0.385^a^	_a_0.384^a^
	Non-genotyped	PBLUP	0.121	–	–	–	–
		ssGBLUP	–	_a_0.191^ab^	_ab_0.192^a^	_ab_0.191^b^	_ab_0.192^ab^
		ssFGBLUP	–	–	_ab_0.194^a^	_b_0.189^c^	_ab_0.191^b^
		ssWGBLUP_bin1	–	_a_0.192^b^	_a_0.194^a^	_a_0.192^b^	_a_0.193^ab^
		ssWGBLUP_bin30	–	_a_0.190^b^	_a_0.194^a^	_a_0.194^a^	_a_0.193^a^
		ssWGBLUP_bin100	–	_a_0.192^a^	_b_0.190^ab^	_b_0.188^c^	_b_0.188^bc^
Protein	Genotyped	PBLUP	0.152	–	–	–	–
		ssGBLUP	–	_b_0.428^c^	_a_0.447^ab^	_a_0.444^b^	_a_0.450^a^
		ssFGBLUP	–	–	_a_0.457^ab^	_a_0.442^b^	_a_0.451^a^
		ssWGBLUP_bin1	–	_a_0.449^ab^	_a_0.452^a^	_a_0.445^b^	_a_0.448^ab^
		ssWGBLUP_bin30	–	_ab_0.445^b^	_a_0.455^a^	_a_0.447^ab^	_a_0.452^ab^
		ssWGBLUP_bin100	–	_ab_0.437^a^	_a_0.443^a^	_a_0.442^a^	_a_0.442^a^
	Non-genotyped	PBLUP	0.140	–	–	–	–
		ssGBLUP	–	_b_0.188^b^	_bc_0.191^a^	_bc_0.190^a^	_b_0.191^a^
		ssFGBLUP	–	–	_bc_0.192^a^	_c_0.188^b^	_bc_0.189^b^
		ssWGBLUP_bin1	–	_a_0.197^a^	_a_0.197^a^	_a_0.194^b^	_a_0.194^b^
		ssWGBLUP_bin30	–	_b_0.190^a^	_b_0.192^a^	_ab_0.192^a^	_b_0.191^a^
		ssWGBLUP_bin100	–	_b_0.191^a^	_c_0.189^a^	_c_0.187^b^	_c_0.187^b^

ssGBLUP models = a single-step genomic BLUP (ssGBLUP) model, a featured ssGBLUP (ssFGBLUP) model, and weighted ssGBLUP models with a region size of 1 (ssWGBLUP_bin1), 30 (ssWGBLUP_bin30), or 100 SNPs (ssWGBLUP_bin100)

SNP sets = SNPs on the 54K-SNP chip (54K), SNPs on the 54K-SNP chip together with sequencing SNPs selected by Denmark-Finland-Sweden (54K + DFS), SNPs on the 54K-SNP chip together with sequencing SNPs selected by France (54K + FRA), and SNPs on the 54K-SNP chip together with both sets of selected sequencing SNPs (54K + DFS + FRA)

_a,b,c_subscript letters to the left are for comparisons among models using the same SNP set, and superscript letters to the right are for comparisons among SNP sets using the same model. Reliabilities with no common letter differ significantly (P < 0.05)

**Table 4:** Reliabilities from a pedigree BLUP (PBLUP) model and different ssGBLUP models using different SNP sets for female fertility traits

Trait	Population	Model	Pedigree	SNP			
				54K	54K + DFS	54K + FRA	54K + DFS + FRA
IFLh	Genotyped	PBLUP	0.318	–	–	–	–
		ssGBLUP	–	_a_0.407^a^	_a_0.398^a^	_a_0.404^a^	_a_0.395^a^
		ssFGBLUP	–	–	_a_0.401^a^	_a_0.387^a^	_a_0.369^a^
		ssWGBLUP_bin1	–	_a_0.406^a^	_a_0.398^a^	_a_0.405^a^	_a_0.396^a^
		ssWGBLUP_bin30	–	_a_0.412^a^	_a_0.398^a^	_a_0.401^a^	_a_0.389^a^
		ssWGBLUP_bin100	–	_a_0.410^a^	_a_0.405^a^	_a_0.405^a^	_a_0.406^a^
	Non-genotyped	PBLUP	0.126	–	–	–	–
		ssGBLUP	–	_a_0.139^a^	_a_0.138^a^	_a_0.140^a^	_a_0.139^a^
		ssFGBLUP	–	–	_a_0.138^a^	_a_0.141^a^	_a_0.138^a^
		ssWGBLUP_bin1	–	_a_0.140^a^	_a_0.137^a^	_a_0.139^a^	_a_0.139^a^
		ssWGBLUP_bin30	–	_a_0.138^a^	_a_0.136^a^	_a_0.139^a^	_a_0.139^a^
		ssWGBLUP_bin100	–	_a_0.139^a^	_a_0.137^a^	_a_0.140^a^	_a_0.143^a^
IFLc	Genotyped	PBLUP	0.086	–	–	–	–
		ssGBLUP	–	_a_0.234^a^	_a_0.229^a^	_a_0.236^a^	_a_0.230^a^
		ssFGBLUP	–	–	_a_0.213^a^	_a_0.234^a^	_a_0.218^a^
		ssWGBLUP_bin1	–	_a_0.239^a^	_a_0.232^a^	_a_0.238^a^	_a_0.234^a^
		ssWGBLUP_bin30	–	_a_0.245^a^	_a_0.239^a^	_a_0.247^a^	_a_0.236^a^
		ssWGBLUP_bin100	–	_a_0.240^a^	_a_0.233^a^	_a_0.244^a^	_a_0.240^a^
	Non-genotyped	PBLUP	0.108	–	–	–	–
		ssGBLUP	–	_a_0.088^a^	_a_0.087^a^	_ab_0.090^a^	_a_0.089^a^
		ssFGBLUP	–	–	_a_0.083^b^	_a_0.093^a^	_a_0.089^ab^
		ssWGBLUP_bin1	–	_a_0.086^a^	_a_0.085^a^	_ab_0.089^a^	_a_0.087^a^
		ssWGBLUP_bin30	–	_a_0.085^a^	_a_0.083^a^	_b_0.086^a^	_a_0.084^a^
		ssWGBLUP_bin100	–	_a_0.084^a^	_a_0.083^a^	_ab_0.084^a^	_a_0.083^a^
ICF	Genotyped	PBLUP	0.031	–	–	–	–
		ssGBLUP	–	_a_0.140^a^	_a_0.143^a^	_a_0.143^a^	_a_0.145^a^
		ssFGBLUP	–	–	_a_0.147^a^	_a_0.149^a^	_a_0.153^a^
		ssWGBLUP_bin1	–	_a_0.140^a^	_a_0.144^a^	_a_0.142^a^	_a_0.145^a^
		ssWGBLUP_bin30	–	_a_0.138^a^	_a_0.140^a^	_a_0.142^a^	_a_0.144^a^
		ssWGBLUP_bin100	–	_a_0.140^a^	_a_0.141^a^	_a_0.142^a^	_a_0.144^a^
	Non-genotyped	PBLUP	0.069	–	–	–	–
		ssGBLUP	–	_a_0.069^c^	_a_0.070^bc^	_a_0.071^ab^	_a_0.072^a^
		ssFGBLUP	–	–	_a_0.073^a^	_a_0.076^a^	_a_0.078^a^
		ssWGBLUP_bin1	–	_a_0.069^b^	_a_0.070^ab^	_a_0.071^a^	_a_0.072^a^
		ssWGBLUP_bin30	–	_a_0.069^b^	_a_0.070^b^	_a_0.071^ab^	_a_0.072^a^
		ssWGBLUP_bin100	–	_a_0.069^b^	_a_0.071^ab^	_a_0.072^a^	_a_0.073^a^

ssGBLUP models = a single-step genomic BLUP (ssGBLUP) model, a featured ssGBLUP (ssFGBLUP) model, and weighted ssGBLUP models with a region size of 1 (ssWGBLUP_bin1), 30 (ssWGBLUP_bin30), or 100 SNPs (ssWGBLUP_bin100)

SNP sets = SNPs on the 54K-SNP chip (54K), SNPs on the 54K-SNP chip together with sequencing SNPs selected by Denmark-Finland-Sweden (54K + DFS), SNPs on the 54K-SNP chip together with sequencing SNPs selected by France (54K + FRA), and SNPs on the 54K-SNP chip together with both sets of selected sequencing SNPs (54K + DFS + FRA)

*IFLh* the interval from first to last insemination in heifers, *IFLc* the interval from first to last insemination in cows, *ICF* the interval from calving to first insemination

_a,b,c_subscript letters to the left are for comparisons among models using the same SNP set, and superscript letters to the right are for comparisons among SNP sets using the same model. Reliabilities with no common letter differ significantly (P < 0.05)

With the ssGBLUP models, the integration of SNPs selected from sequencing data resulted in statistically significant improvements of the prediction reliabilities for milk yield (0.039 for genotyped animals and 0.007 for non-genotyped animals) and protein yield (0.019 for genotyped animals and 0.003 for non-genotyped animals), a small improvement for fat yield (0.004 for genotyped animals and 0.001 for non-genotyped animals), but no improvement for female fertility traits. For milk and protein yields, the different SNP sets including SNPs selected from sequencing data had similar performances in terms of reliability. For example, for genotyped animals, the improvements of reliabilities from integrating SNPs selected from sequencing data ranged from 0.026 with the 54K + FRA SNP set to 0.032 with the 54K + DFS + FRA SNP set.

When using the 54K SNP sets, different single-step genomic prediction models yielded significant differences in reliabilities for milk and protein yields, but no significant difference for fat yield and female fertility traits. When using the 54K + DFS + FRA SNP set, no significant difference in reliability was observed between all the ssGBLUP models for both genotyped and non-genotyped animals, except for fat and protein yields for which the ssWGBLUP models outperformed the basic ssGBLUP model. The average reliabilities over all traits and all SNP sets for the ssWGBLUP models with a region size of 1, 30, 100 SNPs, or considering the whole genome as one region were equal to 0.361, 0.363, 0.360, and 0.358 for genotyped animals, and 0.149, 0.148, 0.147, and 0.148 for non-genotyped animals. Although the differences were very small between region sizes, a region size of 30 SNPs was generally optimal for the ssWGBLUP models, which was consistent with the predictions using a BayesN0 model with different region sizes. For the ssFGBLUP models, the three SNP sets that included SNPs selected from sequencing data were used and compared to the basic ssGBLUP model, no improvement in prediction reliability was observed except for milk production traits with the 54K + DFS SNP set, but this improvement was not significant.

Regression coefficients from the Bayesian models for genotyped animals are in Table S4 (see Additional file 1: Table S4). Compared with the BayesN0_WG model, the BayesN0 models with a region size of 1, 30, or 100 SNPs resulted in less bias (the deviation of regression coefficient from 1) for fat yield, IFLh, and IFLc, more bias for milk yield and ICF, and a similar level of bias for protein yield. Regression coefficients obtained by using different SNP sets were generally similar in all BayesN0 models. Regression coefficients from PBLUP and various ssGBLUP models are in Tables 5 and 6. For genotyped animals, compared to the PBLUP models, the ssGBLUP models resulted in less bias for IFLh and ICF, more bias for milk, fat, and protein yields, and a similar level of bias for IFLc. For non-genotyped animals, compared to the PBLUP models, the ssGBLUP models resulted in less bias for IFLc, more bias for protein yield and ICF, and a similar level of bias for milk and fat yields, and IFLh. With the ssGBLUP models, for both milk production and female fertility traits, less bias was observed for non-genotyped animals than for genotyped animals. Differences in regression coefficients estimated with the different SNPs sets were small when using the ssGBLUP models. Compared to the basic ssGBLUP model, the alternative models, ssWGBLUP and ssFGBLUP, resulted in a similar level of bias or in a significantly increased bias for all SNP sets.

**Table 5:** Regression coefficients of yield deviation (YD) on prediction from a pedigree-based BLUP (PBLUP) model and different ssGBLUP models using different SNP sets for milk production traits

Trait	Population	Model	Pedigree	SNP			
				54K	54K + DFS	54K + FRA	54K + DFS + FRA
Milk	Genotyped	PBLUP	0.89	–	–	–	–
		ssGBLUP	–	_a_0.84^a^	_a_0.83^a^	_a_0.83^a^	_a_0.83^a^
		ssFGBLUP	–	–	_a_0.83^a^	_b_0.81^a^	_a_0.82^a^
		ssWGBLUP_bin1	–	_c_0.80^a^	_b_0.80^a^	_c_0.79^a^	_b_0.80^a^
		ssWGBLUP_bin30	–	_b_0.81^a^	_b_0.80^b^	_bc_0.80^b^	_b_0.80^b^
		ssWGBLUP_bin100	–	_b_0.81^a^	_b_0.81^ab^	_c_0.80^b^	_b_0.80^b^
	Non-genotyped	PBLUP	0.87	–	–	–	–
		ssGBLUP	–	_a_0.87^a^	_a_0.87^a^	_a_0.86^a^	_a_0.86^a^
		ssFGBLUP	–	–	_ab_0.87^a^	_a_0.86^b^	_a_0.86^ab^
		ssWGBLUP_bin1	–	_b_0.85^a^	_c_0.85^a^	_b_0.84^a^	_b_0.85^a^
		ssWGBLUP_bin30	–	_ab_0.85^a^	_c_0.85^a^	_b_0.85^a^	_b_0.85^a^
		ssWGBLUP_bin100	–	_a_0.86^a^	_bc_0.86^a^	^ab^0.85^ab^	_b_0.85^b^
Fat	Genotyped	PBLUP	0.77	–	–	–	–
		ssGBLUP	–	_a_0.74^a^	_a_0.74^a^	_a_0.73^a^	_a_0.73^a^
		ssFGBLUP	–	–	_a_0.75^a^	_a_0.72^b^	_a_0.73^b^
		ssWGBLUP_bin1	–	_b_0.68^a^	_b_0.68^a^	_b_0.68^a^	_b_0.68^a^
		ssWGBLUP_bin30	–	_b_0.69^a^	_b_0.70^a^	_b_0.69^a^	_b_0.70^a^
		ssWGBLUP_bin100	–	_b_0.70^a^	_b_0.69^a^	_b_0.69^a^	_b_0.68^a^
	Non-genotyped	PBLUP	0.77	–	–	–	–
		ssGBLUP	–	_a_0.78^a^	_a_0.78^a^	_a_0.77^b^	_a_0.77^b^
		ssFGBLUP	–	–	_a_0.79^a^	_ab_0.77^b^	_a_0.77^b^
		ssWGBLUP_bin1	–	_b_0.76^a^	_b_0.76^a^	_bc_0.76^a^	_b_0.76^a^
		ssWGBLUP_bin30	–	_b_0.76^b^	_b_0.77^a^	_ab_0.77^a^	_ab_0.76^ab^
		ssWGBLUP_bin100	–	_b_0.76^a^	_b_0.76^ab^	_c_0.75^bc^	_c_0.74^c^
Protein	Genotyped	PBLUP	0.81	–	–	–	–
		ssGBLUP	–	_a_0.76^a^	_a_0.77^a^	_a_0.76^a^	_a_0.76^a^
		ssFGBLUP	–	–	_a_0.78^a^	_a_0.75^b^	_a_0.76^b^
		ssWGBLUP_bin1	–	_b_0.73^a^	_b_0.73^a^	_b_0.72^a^	_b_0.72^a^
		ssWGBLUP_bin30	–	_b_0.73^a^	_b_0.74^a^	_b_0.72^a^	_b_0.73^a^
		ssWGBLUP_bin100	–	_b_0.74^a^	_b_0.73^ab^	_b_0.72^ab^	_b_0.72^b^
	Non-genotyped	PBLUP	0.80	–	–	–	–
		ssGBLUP	–	_a_0.77^a^	_ab_0.77^a^	_a_0.77^a^	_a_0.77^a^
		ssFGBLUP	–	–	_a_0.78^a^	_ab_0.76^b^	_a_0.77^ab^
		ssWGBLUP_bin1	–	_a_0.77^ab^	_ab_0.77^a^	_ab_0.76^ab^	_ab_0.76^b^
		ssWGBLUP_bin30	–	_a_0.76^a^	_b_0.76^a^	_ab_0.76^a^	_a_0.76^a^
		ssWGBLUP_bin100	–	_a_0.77^a^	_b_0.76^ab^	_b_0.75^bc^	_b_0.75^c^

ssGBLUP models = a single-step genomic BLUP (ssGBLUP) model, a featured ssGBLUP (ssFGBLUP) model, and weighted ssGBLUP models with a region size of 1 (ssWGBLUP_bin1), 30 (ssWGBLUP_bin30), or 100 SNPs (ssWGBLUP_bin100)

SNP sets = SNPs on the 54K-SNP chip (54K), SNPs on the 54K-SNP chip together with sequencing SNPs selected by Denmark-Finland-Sweden (54K + DFS), SNPs on the 54K-SNP chip together with sequencing SNPs selected by France (54K + FRA), and SNPs on the 54K-SNP chip together with both sets of selected sequencing SNPs (54K + DFS + FRA)

_a,b,c_subscript letters to the left are for comparisons among models using the same SNP set, and superscript letters to the right are for comparisons among SNP sets using the same model. Regression coefficients with no common letter differ significantly (P < 0.05)

**Table 6:** Regression coefficients of yield deviation (YD) on prediction from a pedigree-based BLUP (PBLUP) model and different ssGBLUP models using different SNP sets for female fertility traits

Trait	Population	Model	Pedigree	SNP			
				54K	54K + DFS	54K + FRA	54K + DFS + FRA
IFLh	Genotyped	PBLUP	1.64	–	–	–	–
		ssGBLUP	–	_a_1.56^a^	_a_1.54^a^	_a_1.55^a^	_a_1.53^a^
		ssFGBLUP	–	–	_a_1.55^a^	_a_1.49^a^	_a_1.46^a^
		ssWGBLUP_bin1	–	_a_1.55^a^	_a_1.54^a^	_a_1.55^a^	_a_1.53^a^
		ssWGBLUP_bin30	–	_a_1.55^a^	_a_1.53^a^	_a_1.53^a^	_a_1.51^a^
		ssWGBLUP_bin100	–	_a_1.54^a^	_a_1.53^a^	_a_1.52^a^	_a_1.53^a^
	Non-genotyped	PBLUP	1.08	–	–	–	–
		ssGBLUP	–	_a_1.10^a^	_a_1.10^a^	_a_1.10^a^	_a_1.10^a^
		ssFGBLUP	–	–	_a_1.02^a^	_a_1.09^a^	_a_1.05^a^
		ssWGBLUP_bin1	–	_a_1.10^a^	_a_1.09^a^	_a_1.10^a^	_a_1.10^a^
		ssWGBLUP_bin30	–	_a_1.10^a^	_a_1.09^a^	_a_1.10^a^	_a_1.10^a^
		ssWGBLUP_bin100	–	_a_1.10^a^	_a_1.09^a^	_a_1.10^a^	_a_1.11^a^
IFLc	Genotyped	PBLUP	1.17	–	–	–	–
		ssGBLUP	–	_a_1.18^a^	_a_1.17^a^	_a_1.20^a^	_a_1.18^a^
		ssFGBLUP	–	–	_a_1.11^a^	_a_1.20^a^	_a_1.14^a^
		ssWGBLUP_bin1	–	_a_1.18^a^	_a_1.16^a^	_a_1.19^a^	_a_1.17^a^
		ssWGBLUP_bin30	–	_a_1.18^a^	_a_1.16^a^	_a_1.19^a^	_a_1.16^a^
		ssWGBLUP_bin100	–	_a_1.17^a^	_a_1.15^a^	_a_1.18^a^	_a_1.17^a^
	Non-genotyped	PBLUP	1.32	–	–	–	–
		ssGBLUP	–	_a_1.03^bc^	_a_1.02^c^	_a_1.05^a^	_a_1.04^ab^
		ssFGBLUP	–	–	_b_0.99^c^	_a_1.07^a^	_ab_1.04^b^
		ssWGBLUP_bin1	–	_b_1.02^b^	_ab_1.01^b^	_b_1.04^a^	_b_1.02^ab^
		ssWGBLUP_bin30	–	_b_1.00^a^	_b_0.99^a^	_c_1.01^a^	_c_1.00^a^
		ssWGBLUP_bin100	–	_b_1.00^a^	_b_0.99^a^	_bc_1.00^a^	_bc_0.99^a^
ICF	Genotyped	PBLUP	0.57	–	–	–	–
		ssGBLUP	–	_a_0.66^a^	_a_0.67^a^	_a_0.67^a^	_a_0.67^a^
		ssFGBLUP	–	–	_a_0.67^a^	_a_0.67^a^	_a_0.68^a^
		ssWGBLUP_bin1	–	_a_0.66^a^	_a_0.67^a^	_a_0.66^a^	_a_0.67^a^
		ssWGBLUP_bin30	–	_a_0.65^a^	_a_0.66^a^	_a_0.66^a^	_a_0.67^a^
		ssWGBLUP_bin100	–	_a_0.65^a^	_a_0.66^a^	_a_0.66^a^	_a_0.66^a^
	Non-genotyped	PBLUP	0.89	–	–	–	–
		ssGBLUP	–	_a_0.71^b^	_a_0.72^ab^	_a_0.73^a^	_a_0.73^a^
		ssFGBLUP	–	–	_a_0.74^a^	_a_0.75^a^	_a_0.76^a^
		ssWGBLUP_bin1	–	_a_0.71^b^	_a_0.72^ab^	_a_0.73^ab^	_a_0.73^a^
		ssWGBLUP_bin30	–	_a_0.71^b^	_a_0.72^ab^	_a_0.73^ab^	_a_0.73^a^
		ssWGBLUP_bin100	–	_a_0.71^b^	_a_0.72^ab^	_a_0.73^a^	_a_0.73^a^

ssGBLUP models = a single-step genomic BLUP (ssGBLUP) model, a featured ssGBLUP (ssFGBLUP) model, and weighted ssGBLUP models with a region size of 1 (ssWGBLUP_bin1), 30 (ssWGBLUP_bin30), or 100 SNPs (ssWGBLUP_bin100)

SNP sets = SNPs in the 54K-SNP chip (54K), SNPs in the 54K-SNP chip together with sequencing SNPs selected by Denmark–Finland–Sweden (54K + DFS), SNPs in the 54K-SNP chip together with sequencing SNPs selected by France (54K + FRA), and SNPs in the 54K-SNP chip together with both sets of selected sequencing SNPs (54K + DFS + FRA)

*IFLh* the interval from first to last insemination in heifers, *IFLc* the interval from first to last insemination in cows, *ICF* the interval from calving to first insemination

_a,b,c_subscript letters to the left are for comparisons among models using the same SNP set, and superscript letters to the right are for comparisons among SNP sets using the same model. Regression coefficients with no common letter differ significantly (P < 0.05)

## Discussion

Most previous studies that investigated the impact of integrating SNPs selected from sequencing data on genomic prediction focused on genotyped animals only [5–7, 33]. In this study, we investigated the effects of integrating such SNPs for both genotyped and non-genotyped animals using ssGBLUP models that make different assumptions on SNP effects. The improvement in prediction reliability from integrating SNPs selected from sequencing data varied according to trait, and the best model also varied according to SNP set and trait.

Integrating SNPs selected from sequencing data improved reliabilities for milk production traits, which have heritabilities of about 0.37 but not for female fertility traits, which have heritabilities lower than 0.05. This is in line with a previous study for genotyped Danish Jersey animals [7], which reported improvements of reliabilities from integrating DFS and FRA SNPs for milk, fat, and protein yields but not for the female fertility index. In Danish Jersey, the selected DFS + FRA SNPs contained more top SNPs for milk production traits than for female fertility traits. For example, among the top 20 SNPs from the 54K + DFS + FRA SNP set for each trait using the BayesN0_bin1 model, nine DFS + FRA SNPs were found for milk yield, four for fat yield, and eight for protein yield, but only one for IFLh, three for IFLc, and one for ICF. Among the three milk production traits, for fat yield there were fewer top SNPs among the SNPs selected from sequencing data and, accordingly, improvement in its reliability from integrating these SNPs was relatively small in Danish Jersey, which was also observed in a previous study [7]. This result could be due to the fact that Jersey cattle are characterized by an extremely high level of fat percentage and have large differences in fat profile compared to other breeds (e.g. Holsteins [34]). Since the SNPs selected from sequencing data were obtained from multiple breeds, they probably have limited effects on fat yield in Danish Jersey, especially when the effect of the *DGAT1* (*diacylglycerol O-acyltransferase *1) gene is already well-accounted for by the 54K-SNP chip (ARS-BFGL-NGS-4939 is the top SNP for fat yield in the 54K-SNP chip and is located within the *DGAT1* gene). For milk production traits, the magnitude of improvement in reliability from integrating SNPs selected from sequencing data was smaller in our study than that reported in [7]. One explanation is that, since the reference population size was larger, the improvement from using an additional source of information, such as integrating SNPs selected from sequencing data, was smaller [7, 35]. In addition, compared with a GBLUP model, the use of pedigree information for both non-genotyped animals and genotyped animals (${\upomega }_{a}=0.2$) in a ssGBLUP model may dilute the impact of integrating SNPs selected from the sequencing data. Compared to the milk production traits, for female fertility traits, the power of QTL detection was expected to be lower due to much lower heritabilities, and the number of SNPs being selected was smaller because of the relatively lower economic importance [7]. For IFLh, we observed slight decreases in reliabilities for genotyped animals after integrating SNPs selected from sequencing data, which could be due to sampling errors resulting from the extremely low heritability of this trait and the relatively small sample size of the validation population (3749 genotyped females with a reliability of YD as low as 0.014). As expected, by using the multiple t-test, none of the decreases in reliability reached the significance threshold. By using a ssGBLUP model, the impact of integrating SNPs selected from sequencing data could be investigated for non-genotyped animals as well. Similar to that observed for genotyped animals, integrating SNPs selected from sequencing data improved reliabilities for milk and protein yields, although the magnitude of improvement was much smaller than that observed for genotyped animals.

The strategy used to select SNPs from sequencing data can highly impact their role in genomic prediction. The DFS and FRA SNPs were selected by association analyses and bioinformatics analyses [6, 20]. The benefits of integrating sequencing SNPs selected by association analyses have been verified in various populations, such as Nordic Holsteins [5], Nordic Red [6], and Danish Jersey [7]. However, very few studies have examined the effect of sequencing SNPs selected by bioinformatics analyses. Fang et al. [36] showed that reliabilities of genomic prediction improved by constructing a model including an additional feature genetic component based on SNPs associated with specific biologically significant Gene Ontology (GO) terms [37], compared to the use of a basic GBLUP model, especially in an across-breed prediction. VanRaden et al. [38] compared strategies of selecting sequencing variants using the sequencing data from the 1000 Bull Genomes project and showed that reliabilities increased slightly (by 0.004) when both 481,904 candidate SNPs selected from association analyses and 249,966 insertions-deletions were integrated into the genotype data of the HD chip. In our study, by integrating the DFS and FRA SNPs that were selected based on sequencing data in multiple breeds, the information from the breeds with large reference population sizes allowed us to improve genomic prediction of Danish Jersey, which is a numerically small population. The importance of using SNPs selected from sequencing data in multiple breeds was previously suggested in a study on Holstein–Friesian bulls [33], which showed that the use of sequencing SNPs selected from single-breed association analyses decreased the prediction reliability and increased the bias for protein yield, somatic cell score, and IFL, because it is difficult to select top SNPs when extended linkage disequilibrium exists within a single breed.

The performance in terms of genomic prediction models depends on the assumptions made about the effects of SNPs, the genetic architecture of the trait, and the SNP set used. In our study, when we used the 54K SNP set, the ssWGBLUP model outperformed the ssGBLUP model for milk and protein yields but not for fat yield and three female fertility traits. This is in line with a previous study that also used the 54K SNP set on genotyped Danish Jersey animals, which showed that a Bayesian R model [15] outperformed the GBLUP model for milk and protein yields, but not for fat yield, mastitis and the female fertility index [7]. Similarly, in French dairy goats, using a 54K-SNP chip, weighted ssGBLUP models outperformed basic ssGBLUP models for traits with identified QTL but not for the traits without known QTL [39]. A similar conclusion was given by Zhang et al. [16] in a simulation study. Tiezzi and Maltecca [40] investigated weighted $\mathbf{G}$ matrices for traits with different genetic architectures in US Holsteins and showed that predictive performances increased and bias decreased for traits with moderate to high heritabilities, such as fat and protein percentages. They also showed that, even for lowly heritable traits such as calving ease, gain in reliability was achieved when using the weighted $\mathbf{G}$ matrix. Other studies reported that the benefit on reliability from using models that prioritize, select, or weight SNPs is greater for small datasets [5, 7]. Compared with the previous study on genotyped Danish Jersey animals [7] that used the 54K SNP set, for genotyped animals, we observed that the benefit on reliability from weighting SNPs decreased for milk and protein yields when a large number of non-genotyped animals were included.

When SNPs selected from sequencing data were integrated in the SNP set, the reliability reached by the best ssWGBLUP model was slightly higher than that from the basic ssGBLUP model for milk and protein yields, but the difference was not statistically significant. In other words, adding SNPs selected from sequencing data to the 54K-SNP chip increased prediction reliability, but no further improvement was obtained by using the weighting strategy. This result does not agree with that of the simulation study by [8], which showed an increase in accuracy by adding causal variants to the standard SNP chip, and a further increase when QTL and SNPs received weights from GWAS. It is possible that the signals of added sequencing SNPs were stolen by the nearby 54K SNPs [38]. In other words, even when the basic ssGBLUP model is used, more weight could be automatically given to the QTL regions for which the marker density is increased by adding SNPs selected from sequencing data. Similar results were also observed in previous studies. In the study on genotyped Danish Jersey animals of [7], the superiority of a Bayesian R model [15] over a GBLUP model was found to decrease after integrating SNPs selected from sequencing data. In another study on eggshell strength, feed intake, and laying rate in a commercial brown layer line using a HD chip and sequencing data, Ni et al. [41] reported that although the highest predictive ability was achieved when using sequencing SNPs that are located in or around a gene, reliability did not increase with a ssWGBLUP model compared to a basic ssGBLUP model. A similar conclusion was drawn by Fragomeni et al. [9], who reported that a basic ssGBLUP model slightly outperformed a ssWGBLUP model for stature in US Holsteins when sequencing SNPs selected by association analyses were integrated in the SNP set.

In the ssWGBLUP models, we used the posterior SNP variances estimated from the BayesN0 models to weight SNPs in the construction of the $\mathbf{G}$ matrices. Previous studies have investigated the weights generated from various parameters, including SNP variances [16, 17, 19, 38, 40, 42–44], squared SNP effects [16–18, 41, 45], and −log_10_(P-values) [17, 41, 45]. Su et al. [17] proved that a GBLUP model with a $\mathbf{G}$ matrix in which the SNPs are weighted by standardized posterior SNP variances estimated from a Bayesian model was theoretically equivalent to a Bayesian model. The superiority of using the posterior variance from Bayesian models compared to other parameters was also confirmed by simulations [16] and analyses of real data [17].

In our study, the weights on SNPs within each region were generated from region-specific variances, which were estimated by a BayesN0 model [31] that provides the variance of SNPs within each non-overlapping genomic region directly. We also applied a ssWGBLUP model in which the weight on SNPs within a region was the average variance of each SNP within the region estimated from a Bayesian R model [15]. Estimating SNP variances first and then generating the weight for each region based on SNP variances is a strategy applied in various studies [17, 18]. Since the performance of the ssWGBLUP model using weights from a Bayesian R model (results not shown) was similar to that observed from a ssWGBLUP model using weights from a BayesN0 model, we present only the results from the latter. By comparing the results obtained with different region sizes, we found that reliabilities varied little with region size, except for protein yield for which a 30-SNP region resulted in a slightly higher reliability than a 1- or 100-SNP region when SNPs selected from sequencing data were integrated. Su et al. [17] compared weighted GBLUP models with a region size of 1, 5, 10, 30, 50, 70, 100 and 150 SNPs using the 54K-SNP chip in Nordic Holsteins and suggested that a region size of 30 SNPs was optimal, but differences between different region sizes were less than 1percentage point. Teissier et al. [19] compared weights from a single-marker weighting strategy with a group-marker weighting strategy in which the weight was either the sum or the maximum of single-marker weights among all SNPs within that region, and showed that the group-marker strategy outperformed the single-marker strategy. The reasoning behind the group-marker strategy was that the weight from the estimated parameter of a single SNP has a large uncertainty compared with the average from a group of SNPs. In addition, many studies applied ssWGBLUP models using an iterative process [18, 19, 42] in which the solutions of the current ssGBLUP model were used to generate quadratic weights (e.g. SNP variance [19] and squared SNP effects [18]) on SNPs in the next run. According to Wang et al. [42] and Teissier et al. [19], the second iteration was optimal if the unweighted ssGBLUP model was considered as the first iteration, since more iterations would cause overweighting of SNPs with large effects and underweighting of SNPs with small effects. The ssWGBLUP model in our study used the variances estimated from the Bayesian model, which avoids the problem with the iterative procedure. Furthermore, under a framework of a single-trait evaluation, the ssWGBLUP model can take advantage of Bayesian models (weights could be lagged for three years based on Su et al. [17]) while keeping a similar computational cost to that of the basic ssGBLUP model.

Similar to the ssWGBLUP model, the ssFGBLUP model, which we introduce for the first time here, was allowed to place more emphasis on the SNPs selected from sequencing data than on the SNPs of the 54K-SNP chip. One difference between the ssFGBLUP and ssWGBLUP models is that ssFGBLUP assumes that all the SNPs within a same genetic component share the same variance, whereas ssWGBLUP allows each SNP or each SNP region to have its own variance and therefore does not prioritize all SNPs selected from sequencing data. In our study, no significant difference was found between the reliabilities from ssFGBLUP and a basic ssGBLUP model. On the one hand, the basic ssGBLUP model automatically places more emphasis on the QTL regions because of the increased marker density in QTL regions due to the addition of SNPs selected from sequencing data, which were more likely to be causal SNPs or highly linked to causal SNPs. On the other hand, in the ssFGBLUP model, the proportions of variances accounted for by the two genetic components (54K SNPs vs. selected sequencing SNPs) were approximately derived from variance components estimated using a FGBLUP model. Such an approximation may be not optimal, because FGBLUP uses $\mathbf{G}$ matrices, which involve only genotyped animals whereas ssFGBLUP uses $\mathbf{H}$ matrices, which involve both genotyped and non-genotyped animals.

Regarding regression coefficients, with the same model, there was little difference among different SNP sets for both genotyped and non-genotyped animals. This was consistent with the finding from a previous study on stature in US Holsteins, where no difference in regression coefficients was observed between 54K and 54K plus 16,648 selected sequencing SNPs for both basic ssGBLUP and ssWGBLUP (with weights derived from squared SNP effects) models [9]. In our study, considering both the results from regression coefficients and reliabilities, the integration of selected sequencing SNPs into the standard SNP chip was suggested for predicting breeding values since it could improve the prediction reliability without compromising unbiasedness. With the same SNP set, we found that the regression coefficients for all milk production traits decreased with ssWGBLUP model compared to the basic ssGBLUP model. Given that all milk production traits had regression coefficients lower than 1 for both genotyped and non-genotyped animals, the use of the weighting strategy as used in ssWGBLUP models increased the inflation of EBV. A previous study on stature in US Holsteins [9] reported similar results i.e., that for both 54K and 54K plus 16,648 selected sequencing SNPs, the regression coefficient of ssGBLUP models decreased from 0.88 to 0.79 after weighting SNPs by squared SNP effects. However, when weighting of the SNPs was carried out by a nonlinear method [12] that resembled the Bayesian A method, the change in regression coefficients was limited. In our study, considering both the results from regression coefficients and reliabilities, a basic ssGBLUP model was suggested when integrating SNPs selected from sequencing data since the ssWGBLUP model resulted in little improvement in reliabilities but could lead to more inflation in EBV. For IFLh and ICF, unstable regression coefficients were observed for genotyped animals, even when using a PBLUP model which did not involve any genomic information. The reasons causing the unusual regression coefficients are unclear. One possible reason for this could be the consequence of the low heritability of the trait (lower than 0.05) and a relatively small sample size of the validation population (3749 for IFLh and 2833 for ICF), where a small change in additive genetic variance resulting from a sampling error could lead to a large change on the EBV scale. However, this may not be sufficient to account for these unusual regression coefficients. There may be some underlying mechanisms that are difficult to identify.

## Conclusions

In summary, we show that when using the genotype data of the 54K-SNP chip, a ssWGBLUP model with a common weight on the SNPs within a specific region (about 30 SNPs) can be a feasible approach for routine genomic evaluation. Integrating relevant SNPs selected from sequencing data by association and bioinformatics analyses into the standard SNP chip slightly improves genomic prediction reliability. With such a SNP set, we recommend the use of a basic ssGBLUP model since no significant improvement is observed from using alternative models such as ssWGBLUP and ssFGBLUP.

## Supplementary information

**Additional file 1: Table S1.** Title: Variance components estimated from the pedigree-based BLUP (PBLUP) model. Description: The data provided variance components estimated from the PBLUP model. **Table S2.** Title: Variance components1 estimated from a featured genomic BLUP (FGBLUP) model, where the selected sequencing SNPs were considered as a feature component. Description: The data provided variance components estimated from the FGBLUP model. **Table S3.** Title: Reliabilities from Bayesian models for genotyped animals. Description: The data provided prediction reliabilities estimated from Bayesian whole-genome regression models using genotyped animals in the reference population. **Table S4. **Title: Regression coefficients of yield deviation (YD) on prediction from Bayesian models for genotyped animals. Description: The data provided regression coefficients of YD on prediction from Bayesian whole-genome regression models for genotyped animals in the reference population.

## Data Availability

Since the datasets analysed during the current study were generated from commercial dairy farms, they are not publicly available.

## References

[1] Daetwyler HD, Capitan A, Pausch H, Stothard P, van Binsbergen R, Brøndum RF (2014). Whole-genome sequencing of 234 bulls facilitates mapping of monogenic and complex traits in cattle. Nat Genet.

[2] Mao X, Sahana G, De Koning DJ, Guldbrandtsen B (2016). Genome-wide association studies of growth traits in three dairy cattle breeds using whole-genome sequence data. J Anim Sci.

[3] Michot P, Chahory S, Marete A, Grohs C, Dagios D, Donzel E (2016). A reverse genetic approach identifies an ancient frameshift mutation in RP1 causing recessive progressive retinal degeneration in European cattle breeds. Genet Sel Evol.

[4] Boussaha M, Esquerré D, Barbieri J, Djari A, Pinton A, Letaief R (2015). Genome-wide study of structural variants in bovine Holstein, Montbeliarde and Normande dairy breeds. PLoS One.

[5] Ma P, Lund MS, Aamand GP, Su G (2019). Use of a Bayesian model including QTL markers increases prediction reliability when test animals are distant from the reference population. J Dairy Sci.

[6] Brøndum RF, Su G, Janss L, Sahana G, Guldbrandtsen B, Boichard D (2015). Quantitative trait loci markers derived from whole genome sequence data increases the reliability of genomic prediction. J Dairy Sci.

[7] Liu A, Lund MS, Boichard D, Karaman E, Fritz S, Aamand GP (2020). Improvement of genomic prediction by integrating additional single nucleotide polymorphisms selected from imputed whole genome sequencing data. Heredity (Edinb).

[8] Fragomeni BO, Lourenco DAL, Masuda Y, Legarra A, Misztal I (2017). Incorporation of causative quantitative trait nucleotides in single-step GBLUP. Genet Sel Evol.

[9] Fragomeni BO, Lourenco DA, Legarra A, VanRaden PM, Misztal I (2019). Alternative SNP weighting for single-step genomic best linear unbiased predictor evaluation of stature in US Holsteins in the presence of selected sequence variants. J Dairy Sci.

[10] Legarra A, Aguilar I, Misztal I (2009). A relationship matrix including full pedigree and genomic information. J Dairy Sci.

[11] Christensen OF, Lund MS (2010). Genomic prediction when some animals are not genotyped. Genet Sel Evol.

[12] VanRaden PM (2008). Efficient methods to compute genomic predictions. J Dairy Sci.

[13] Meuwissen THE, Hayes BJ, Goddard ME (2001). Prediction of total genetic value using genome-wide dense marker maps. Genetics.

[14] Garrick D, Dekkers J, Fernando R (2014). The evolution of methodologies for genomic prediction. Livest Sci.

[15] Erbe M, Hayes BJ, Matukumalli LK, Goswani S, Bowman PJ, Reich CM, Mason BA, Goddard ME (2012). Improving accuracy of genomic predictions within and between dairy cattle breeds with high density SNP panels. J Dairy Sci..

[16] Zhang Z, Liu J, Ding X, Bijma P, de Koning DJ, Zhang Q (2010). Best linear unbiased prediction of genomic breeding values using a trait-specific marker-derived relationship matrix. PLoS One.

[17] Su G, Christensen OF, Janss L, Lund MS (2014). Comparison of genomic predictions using genomic relationship matrices built with different weighting factors to account for locus-specific variances. J Dairy Sci.

[18] Zhang X, Lourenco D, Aguilar I, Legarra A, Misztal I (2016). Weighting strategies for single-step genomic BLUP: an iterative approach for accurate calculation of GEBV and GWAS. Front Genet.

[19] Teissier M, Larroque H, Robert-Granié C (2018). Weighted single-step genomic BLUP improves accuracy of genomic breeding values for protein content in French dairy goats: a quantitative trait influenced by a major gene. Genet Sel Evol.

[20] Boichard D, Boussaha M, Capitan A, Rocha D, Hozé C, Sanchez MP, et al. Experience from large scale use of the EuroGenomics custom SNP chip in cattle. In: Proceedings of the 11th World Congress on Genetics Applied to Livestock Production, 11–16 February 2018; Auckland. 2018.

[21] McLaren W, Gil L, Hunt SE, Riat HS, Ritchie GR, Thormann A (2016). The Ensembl variant effect predictor. Genome Biol.

[22] Boichard D, Chung H, Dassonneville R, David X, Eggen A, Fritz S (2012). Design of a bovine low-density SNP array optimized for imputation. PLoS One.

[23] Liu A, Lund MS, Boichard D, Mao X, Karaman E, Fritz S (2020). Imputation for sequencing variants preselected to a customized low-density chip. Sci Rep.

[24] Sargolzaei M, Chesnais JP, Schenkel FS (2014). A new approach for efficient genotype imputation using information from relatives. BMC Genomics.

[25] Mäntysaari EA. Combining test day and full lactation records in prediction of breeding values. In: Proceedings of the 7th World Congress on Genetics Applied to Livestock Production: 19–23 August 2002; Montpellier. 2002.

[26] NAV. NAV routine genetic evaluation of dairy cattle. In: Data and genetic models. 2018. https://www.nordicebv.info/wp-content/uploads/2019/02/NAV-routine-genetic-evaluation-2019.pdf. Accessed 29 Aug 2019.

[27] Henderson CR (1984). Application of linear models in animal breeding.

[28] Madsen P, Jensen J. A user’s guide to DMU. Version 6, release 5.1. 2012.

[29] Gao H, Christensen OF, Madsen P, Nielsen US, Zhang Y, Lund MS (2012). Comparison on genomic predictions using three GBLUP methods and two single-step blending methods in the Nordic Holstein population. Genet Sel Evol.

[30] Christensen OF, Madsen P, Nielsen B, Ostersen T, Su G (2012). Single-step methods for genomic evaluation in pigs. Animal.

[31] Zeng J, Garrick D, Dekkers J, Fernando R (2018). A nested mixture model for genomic prediction using whole-genome SNP genotypes. PLoS One.

[32] Bezanson J, Edelman A, Karpinski S, Shah VB (2017). Julia: a fresh approach to numerical computing. SIAM Rev.

[33] Veerkamp RF, Bouwman AC, Schrooten C, Calus MP (2016). Genomic prediction using preselected DNA variants from a GWAS with whole-genome sequence data in Holstein-Friesian cattle. Genet Sel Evol.

[34] Poulsen NA, Gustavsson F, Glantz M, Paulsson M, Larsen LB, Larsen MK (2012). The influence of feed and herd on fatty acid composition in 3 dairy breeds (Danish Holstein, Danish Jersey, and Swedish Red). J Dairy Sci.

[35] Daetwyler HD, Xiang R, Yuan Z, Bolormaa S, Vander Jagt CJ, Hayes BJ, et al. Integration of functional genomics and phenomics into genomic prediction raises its accuracy in sheep and dairy cattle. In: 23rd Conference on Proceedings of the Association for the Advancement of Animal Breeding and Genetics: 27 October to 1 November 2019; Armidale. 2019.

[36] Fang L, Sahana G, Ma P, Su G, Yu Y, Zhang S (2017). Use of biological priors enhances understanding of genetic architecture and genomic prediction of complex traits within and between dairy cattle breeds. BMC Genomics.

[37] Ashburner M, Ball CA, Blake JA, Botstein D, Butler H, Cherry JM (2000). Gene ontology: tool for the unification of biology. Nat Genet.

[38] VanRaden PM, Tooker ME, O’Connell JR, Cole JB, Bickhart DM (2017). Selecting sequence variants to improve genomic predictions for dairy cattle. Genet Sel Evol.

[39] Teissier M, Larroque H, Robert-Granie C (2019). Accuracy of genomic evaluation with weighted single-step genomic best linear unbiased prediction for milk production traits, udder type traits, and somatic cell scores in French dairy goats. J Dairy Sci.

[40] Tiezzi F, Maltecca C (2015). Accounting for trait architecture in genomic predictions of US Holstein cattle using a weighted realized relationship matrix. Genet Sel Evol.

[41] Ni G, Cavero D, Fangmann A, Erbe M, Simianer H (2017). Whole-genome sequence-based genomic prediction in laying chickens with different genomic relationship matrices to account for genetic architecture. Genet Sel Evol.

[42] Wang H, Misztal I, Aguilar I, Legarra A, Mui WM (2012). Genome-wide association mapping including phenotypes from relatives without genotypes. Genet Res.

[43] Lourenco DAL, Fragomeni BO, Bradford HL, Menezes IR, Ferraz JBS, Aguilar I (2017). Implications of SNP weighting on single-step genomic predictions for different reference population sizes. J Anim Breed Genet.

[44] Karaman E, Lund MS, Anche MT, Janss L, Su G (2018). Genomic prediction using multi-trait weighted GBLUP accounting for heterogeneous variances and covariances across the genome. G3 (Bethesda).

[45] de los Campos G, Vazquez AI, Fernando R, Klimentidis YC, Sorensen D (2013). Prediction of complex human traits using the genomic best linear unbiased predictor. PLoS Genet..

