# Supplementary material for: Weighted single-step genomic best linear unbiased prediction integrating variants selected from sequencing data by association and bioinformatics analyses
Source: Genet Sel Evol. 2020 Aug 14;52:48. doi: 10.1186/s12711-020-00568-0 (PMC7429790; doi:10.1186/s12711-020-00568-0)
Supplement: Supplementary file 1 — Additional file 1: Table S1. Title: Variance components estimated from the pedigree-based BLUP (PBLUP) model. Description: The data provided variance components estimated from the PBLUP model. Table S2. Title: Variance components1 estimated from a featured genomic BLUP (FGBLUP) model, where the selected sequencing SNPs were considered as a feature component. Description: The data provided variance components estimated from the FGBLUP model. Table S3. Title: Reliabilities from Bayesian models for genotyped animals. Description: The data provided prediction reliabilities estimated from Bayesian whole-genome regression models using genotyped animals in the reference population. Table S4. Title: Regression coefficients of yield deviation (YD) on prediction from Bayesian models for genotyped animals. Description: The data provided regression coefficients of YD on prediction from Bayesian whole-genome regression models for genotyped animals in the reference population. [file 12711_2020_568_MOESM1_ESM.docx]

Table S1: Variance components^1^ estimated from the pedigree-based BLUP (PBLUP) model.

| Trait^2^ | $\sigma_{a}^{2}$ | $\sigma_{pe}^{2}$ | $\sigma_{hys}^{2}$ | $\sigma_{e}^{2}$ |
| --- | --- | --- | --- | --- |
| Milk | 336,743.9 | 195,653.5 | 95,454.3 | 499,993.5 |
| Fat | 677.3 | 575.5 | 362.5 | 1,649.5 |
| Protein | 383.3 | 310.8 | 165.8 | 762.5 |
| IFLh | 23.9 | -- | 59.6 | 1,796.6 |
| IFLc | 79.8 | 191.5 | 45.6 | 3,002.3 |
| ICF | 36.2 | 42.7 | 73.7 | 782.1 |

^1^Variance components are the additive genetic variance ($\sigma_{a}^{2}$), the permanent environmental variance ($\sigma_{pe}^{2}$), the variance explained by the herd-year-season effect ($\sigma_{hys}^{2}$), and the residual variance ($\sigma_{e}^{2}$).

^2^Traits are the interval from first to last insemination in heifers (IFLh) and cows (IFLc), and the interval from calving to first insemination (ICF).

Table S2: Variance components^1^ estimated from a featured genomic BLUP (FGBLUP) model, where the selected sequencing SNPs were considered as a feature component.

| Trait^2^ | Feature^3^ | $\sigma_{a_{54K}}^{2}$ | $\sigma_{a_{seq}}^{2}$ | $\sigma_{hys}^{2}$ | $\sigma_{pe}^{2}$ | $\sigma_{e}^{2}$ |
| --- | --- | --- | --- | --- | --- | --- |
| Milk | DFS | 240,081.4 | 89,879.4 | 198,750.6 | 95,146.7 | 497,236.2 |
|  | FRA | 214,426.1 | 116,581.3 | 198,265.4 | 95,101.2 | 497,226.7 |
|  | DFS+FRA | 192,814.2 | 137,510.4 | 198,505.4 | 95,126.1 | 497,234.7 |
| Fat | DFS | 565.7 | 99.9 | 586.9 | 361.9 | 1,639.8 |
|  | FRA | 552.0 | 113.8 | 586.8 | 361.8 | 1,639.8 |
|  | DFS+FRA | 505.2 | 160.4 | 586.9 | 361.9 | 1,639.8 |
| Protein | DFS | 305.9 | 68.6 | 317.8 | 165.5 | 757.7 |
|  | FRA | 284.2 | 90.4 | 317.8 | 165.5 | 757.7 |
|  | DFS+FRA | 257.6 | 116.7 | 317.8 | 165.5 | 757.7 |
| IFLh | DFS | 14.64 | 10.52 | 62.97 | -- | 1,817.85 |
|  | FRA | 16.54 | 8.53 | 62.95 | -- | 1,817.95 |
|  | DFS+FRA | 10.91 | 14.19 | 62.95 | -- | 1,817.90 |
| IFLc | DFS | 72.24 | 8.84 | 181.87 | 49.99 | 3,028.6 |
|  | FRA | 69.41 | 11.46 | 182.01 | 49.99 | 3,028.6 |
|  | DFS+FRA | 63.21 | 17.83 | 181.9 | 49.99 | 3,028.6 |
| ICF | DFS | 32.00 | 5.17 | 53.30 | 63.34 | 780.82 |
|  | FRA | 25.83 | 11.38 | 43.11 | 73.50 | 780.83 |
|  | DFS+FRA | 22.82 | 14.38 | 43.12 | 73.50 | 780.83 |

^1^Variance components are the variance explained by SNPs in the 54K chip ($\sigma_{a_{54K}}^{2}$), the variance explained by the feature component ($\sigma_{a_{seq}}^{2}$), the permanent environmental variance ($\sigma_{pe}^{2}$), the variance explained by the herd-year-season effect ($\sigma_{hys}^{2}$), and the residual variance ($\sigma_{e}^{2}$).

^2^Traits are the interval from first to last insemination in heifers (IFLh) and cows (IFLc), and the interval from calving to first insemination (ICF).

^3^The feature component is constructed by sequencing SNPs selected by Denmark-Finland-Sweden (DFS), by France (FRA), or by both Denmark-Finland-Sweden and France (DFS + FRA).

Table S3: Reliabilities from Bayesian models^1^ for genotyped animals.

| Trait^3^ | Model | SNP^4^ | | | |
| --- | --- | --- | --- | --- | --- |
|  |  | 54K | 54K + DFS | 54K + FRA | 54K + DFS + FRA |
| Milk | BayesN0_bin1 | _ab_0.621^a^ | _ab_0.626^a^ | _ab_0.626^a^ | _ab_0.629^a^ |
|  | BayesN0_bin30 | _a_0.628^b^ | _a_0.634^a^ | _a_0.631^ab^ | _a_0.634^ab^ |
|  | BayesN0_bin100 | _b_0.615^b^ | _ab_0.627^a^ | _ab_0.628^a^ | _ab_0.630^a^ |
|  | BayesN0_WG | _c_0.579^c^ | _b_0.618^ab^ | _b_0.618^b^ | _b_0.624^a^ |
| Fat | BayesN0_bin1 | _b_0.359^a^ | _b_0.362^a^ | _b_0.360^a^ | _b_0.363^a^ |
|  | BayesN0_bin30 | _ab_0.363^a^ | _ab_0.365^a^ | _ab_0.366^a^ | _ab_0.368^a^ |
|  | BayesN0_bin100 | _ab_0.358^a^ | _b_0.356^a^ | _ab_0.358^a^ | _b_0.358^a^ |
|  | BayesN0_WG | _a_0.376^ab^ | _a_0.381^ab^ | _a_0.376^b^ | _a_0.381^a^ |
| Protein | BayesN0_bin1 | _a_0.444^a^ | _ab_0.446^a^ | _a_0.442^a^ | _ab_0.444^a^ |
|  | BayesN0_bin30 | _a_0.443^a^ | _a_0.446^a^ | _a_0.442^a^ | _a_0.445^a^ |
|  | BayesN0_bin100 | _b_0.423^a^ | _b_0.431^a^ | _a_0.433^a^ | _b_0.433^a^ |
|  | BayesN0_WG | _b_0.421^c^ | _ab_0.439^ab^ | _a_0.437^b^ | _ab_0.444^a^ |
| IFlh | BayesN0_bin1 | _a_0.297^a^ | _a_0.281^a^ | _a_0.300^a^ | _a_0.290^a^ |
|  | BayesN0_bin30 | _a_0.306^a^ | _a_0.283^a^ | _a_0.285^a^ | _a_0.275^a^ |
|  | BayesN0_bin100 | _a_0.286^a^ | _a_0.267^a^ | _a_0.301^a^ | _a_0.288^a^ |
|  | BayesN0_WG | _a_0.295^a^ | _a_0.297^a^ | _a_0.290^a^ | _a_0.303^a^ |
| IFlc | BayesN0_bin1 | _a_0.204^a^ | _a_0.198^a^ | _a_0.207^a^ | _a_0.204^a^ |
|  | BayesN0_bin30 | _a_0.209^a^ | _a_0.212^a^ | _a_0.217^a^ | _a_0.210^a^ |
|  | BayesN0_bin100 | _a_0.214^a^ | _a_0.210^a^ | _a_0.215^a^ | _a_0.216^a^ |
|  | BayesN0_WG | _a_0.188^a^ | _a_0.182^a^ | _a_0.193^a^ | _a_0.202^a^ |
| ICF | BayesN0_bin1 | _a_0.046^a^ | _a_0.046^a^ | _a_0.051^a^ | _a_0.052^a^ |
|  | BayesN0_bin30 | _a_0.045^a^ | _a_0.044^a^ | _a_0.053^a^ | _a_0.050^a^ |
|  | BayesN0_bin100 | _a_0.045^a^ | _a_0.046^a^ | _a_0.052^a^ | _a_0.051^a^ |
|  | BayesN0_WG | _a_0.032^a^ | _a_0.027^a^ | _a_0.034^a^ | _a_0.033^a^ |

^1^BayesN0 models with a region size of 1 (BayesN0_bin1), 30 (BayesN0_bin30), 100 SNPs (BayesN0_bin100), or the whole genome (BayesN0_WG, equivalent to GBLUP).

^2^Traits are the interval from first to last insemination in heifers (IFLh) and cows (IFLc), and the interval from calving to first insemination (ICF).

^3^The SNP sets are SNPs in the 54K chip (54K), SNPs in the 54K chip together with sequencing SNPs selected by Denmark-Finland-Sweden (54K + DFS), SNPs in the 54K chip together with sequencing SNPs selected by France (54K + FRA), and SNPs in the 54K chip together with both sets of selected sequencing SNPs (54K + DFS + FRA).

^a,b,c^Letters in the left subscripts are for comparisons among models using the same SNP set, and letters in the right superscripts are for comparisons among SNP sets using the same model. Reliabilities with no common letter differ significantly (P < 0.05).

Table S4: Regression coefficients of yield deviation (YD) on prediction from Bayesian models^1^ for genotyped animals.

| Trait^2^ | Model | SNP^3^ | | | |
| --- | --- | --- | --- | --- | --- |
|  |  | 54K | 54K+DFS | 54K+FRA | 54K+DFS+FRA |
| Milk | BayesN0_bin1 | _c_0.91^a^ | _d_0.91^a^ | _d_0.91^a^ | _c_0.91^a^ |
|  | BayesN0_bin30 | _b_0.94^a^ | _c_0.94^a^ | _c_0.94^a^ | _b_0.94^a^ |
|  | BayesN0_bin100 | _b_0.94^b^ | _b_0.96^a^ | _b_0.95^ab^ | _b_0.95^ab^ |
|  | BayesN0_WG | _a_0.97^a^ | _a_0.98^a^ | _a_0.98^a^ | _a_0.98^a^ |
| Fat | BayesN0_bin1 | _a_1.04^a^ | _a_1.05^a^ | _a_1.04^a^ | _a_1.05^a^ |
|  | BayesN0_bin30 | _b_0.98^a^ | _b_0.98^a^ | _b_0.99^a^ | _b_0.99^a^ |
|  | BayesN0_bin100 | _c_0.94^a^ | _c_0.94^a^ | _c_0.94^a^ | _c_0.93^a^ |
|  | BayesN0_WG | _d_0.90^a^ | _c_0.91^a^ | _d_0.90^a^ | _c_0.91^a^ |
| Protein | BayesN0_bin1 | _a_1.05^a^ | _a_1.05^a^ | _a_1.04^a^ | _a_1.04^a^ |
|  | BayesN0_bin30 | _b_1.02^a^ | _b_1.03^a^ | _b_1.02^a^ | _b_1.02^a^ |
|  | BayesN0_bin100 | _c_0.99^a^ | _c_0.99^a^ | _c_0.99^a^ | _c_0.99^a^ |
|  | BayesN0_WG | _c_0.96^a^ | _c_0.97^a^ | _d_0.96^a^ | _c_0.97^a^ |
| IFlh | BayesN0_bin1 | _b_1.40^a^ | _b_1.37^a^ | _a_1.41^a^ | _b_1.38^a^ |
|  | BayesN0_bin30 | _b_1.43^a^ | _b_1.37^a^ | _ab_1.38^a^ | _b_1.35^a^ |
|  | BayesN0_bin100 | _b_1.40^a^ | _b_1.36^a^ | _ab_1.43^a^ | _b_1.39^a^ |
|  | BayesN0_WG | _a_1.62^b^ | _a_1.74^ab^ | _b_1.32^c^ | _a_1.85^a^ |
| IFlc | BayesN0_bin1 | _d_1.01^a^ | _c_0.98^a^ | _c_1.02^a^ | _c_1.00^a^ |
|  | BayesN0_bin30 | _c_1.07^a^ | _b_1.07^a^ | _b_1.10^a^ | _b_1.07^a^ |
|  | BayesN0_bin100 | _b_1.14^a^ | _b_1.12^a^ | _b_1.14^a^ | _b_1.13^a^ |
|  | BayesN0_bWG | _a_1.64^a^ | _a_1.56^b^ | _a_1.56^ab^ | _a_1.62^ab^ |
| ICF | BayesN0_bin1 | _a_0.44^a^ | _a_0.44^a^ | _a_0.46^a^ | _a_0.47^a^ |
|  | BayesN0_bin30 | _a_0.46^a^ | _a_0.46^a^ | _a_0.50^a^ | _a_0.49^a^ |
|  | BayesN0_bin100 | _a_0.50^a^ | _a_0.50^a^ | _a_0.53^a^ | _a_0.52^a^ |
|  | BayesN0_WG | _a_0.79^a^ | _a_0.77^a^ | _a_0.84^a^ | _a_0.85^a^ |

^1^BayesN0 models with a region size of 1 (BayesN0_bin1), 30 (BayesN0_bin30), 100 SNPs (BayesN0_bin100), or the whole genome (BayesN0_WG, equivalent to GBLUP).

^2^Traits are the interval from first to last insemination in heifers (IFLh) and cows (IFLc), and the interval from calving to first insemination (ICF).

^3^The SNP sets are SNPs in the 54K chip (54K), SNPs in the 54K chip together with sequencing SNPs selected by Denmark-Finland-Sweden (54K + DFS), SNPs in the 54K chip together with sequencing SNPs selected by France (54K + FRA), and SNPs in the 54K chip together with both sets of selected sequencing SNPs (54K + DFS + FRA).

^a,b,c,d^Letters in the left subscripts are for comparisons among models using the same SNP set, and letters in the right superscripts are for comparisons among SNP sets using the same model. Regression coefficients with no common letter differ significantly (P < 0.05).
